# Supplementary material for: Unveiling the microbial realm with VEBA 2.0: a modular bioinformatics suite for end-to-end genome-resolved prokaryotic, (micro)eukaryotic and viral multi-omics from either short- or long-read sequencing
Source: Nucleic Acids Res. 2024 Jun 22;52(14):e63. doi: 10.1093/nar/gkae528 (PMC11317156; doi:10.1093/nar/gkae528)
Supplement: gkae528_Supplemental_Files [file gkae528_supplemental_files.zip › VEBA-v2_Supplementary-Material__Revised.pdf]

Supplementary Figures

A

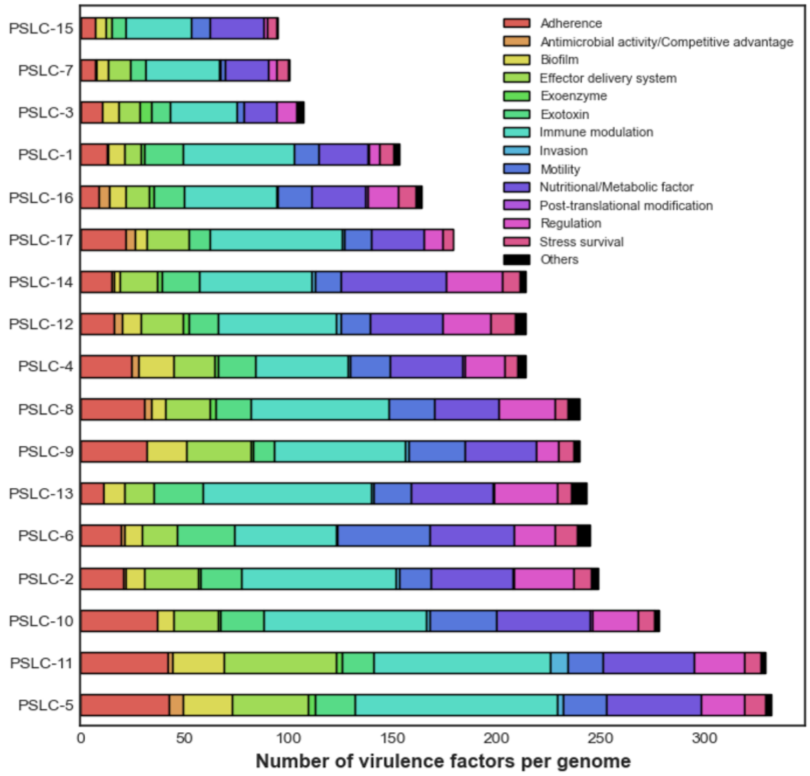

B

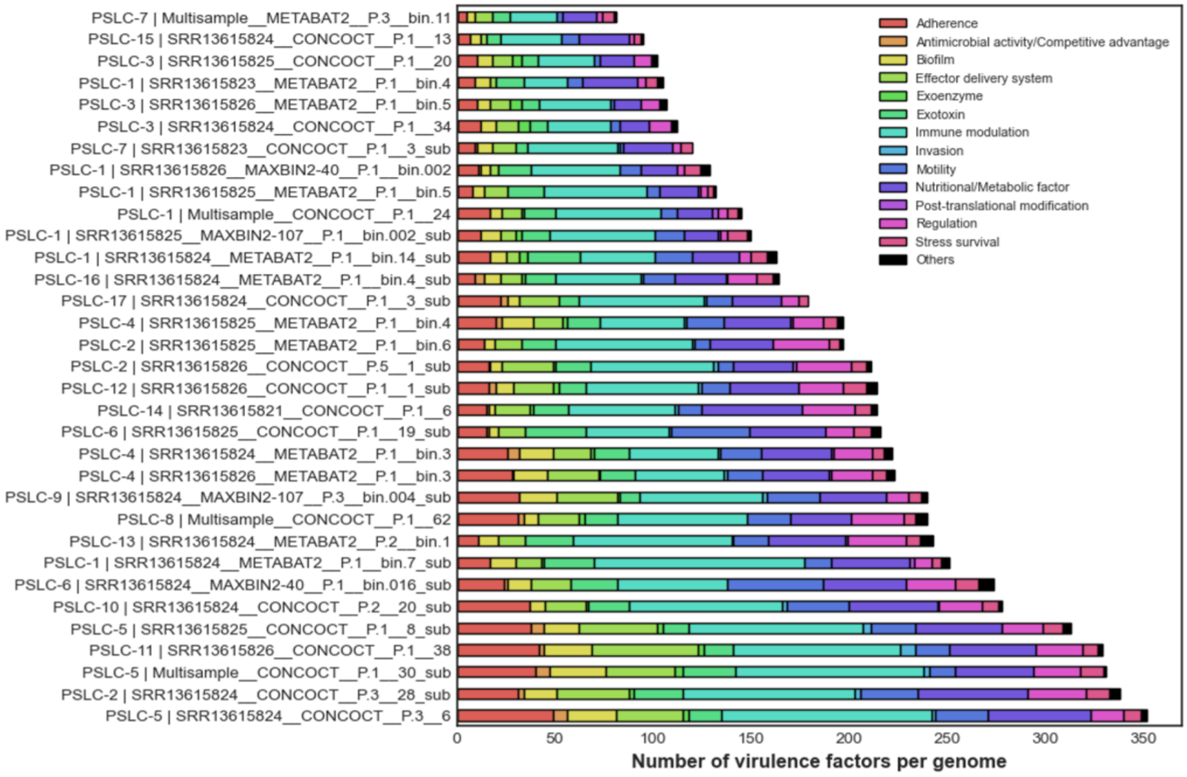

Figure S1 – Distribution of virulence factors in permafrost prokaryotes

Number of virulence factor genes grouped by virulence category for (A) each SLC per genome and (B) each genome.

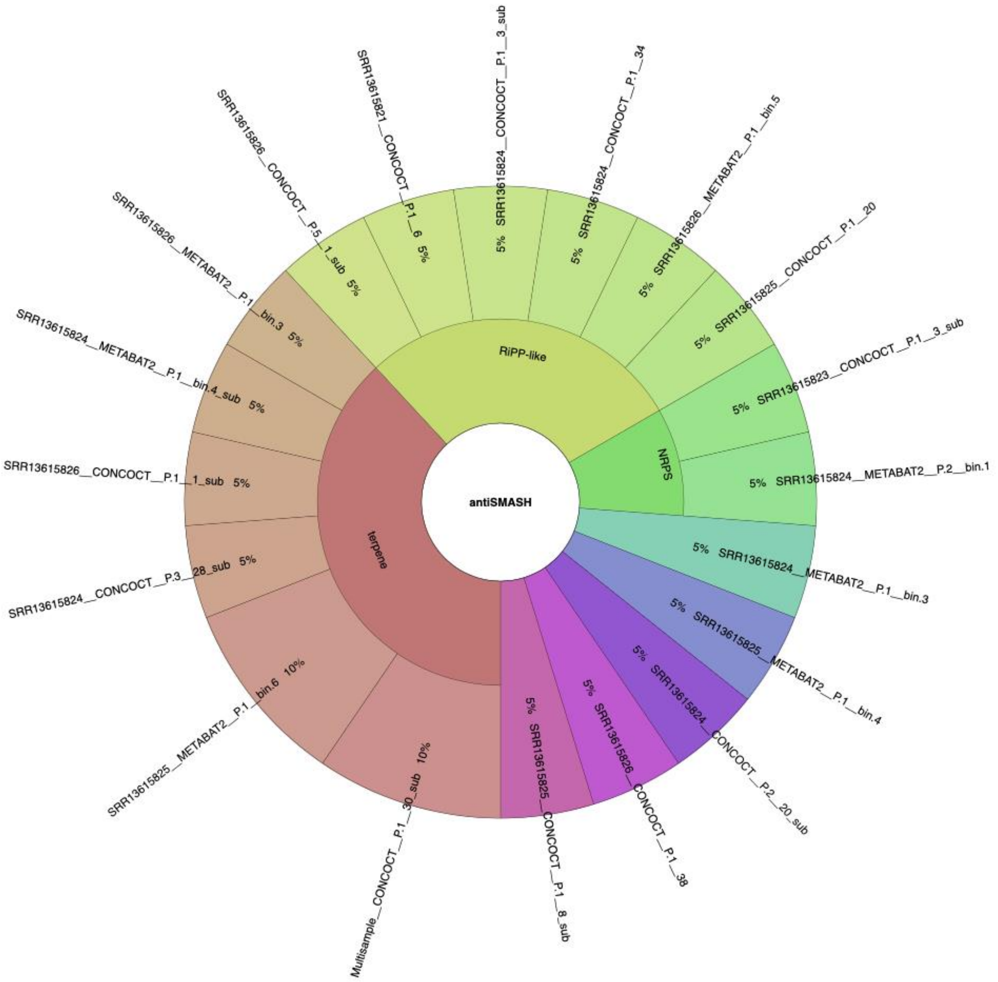

Figure S2 – Distribution of biosynthetic gene clusters in permafrost prokaryotes

Distribution of biosynthetic gene clusters identified by *antiSMASH* for each genome.

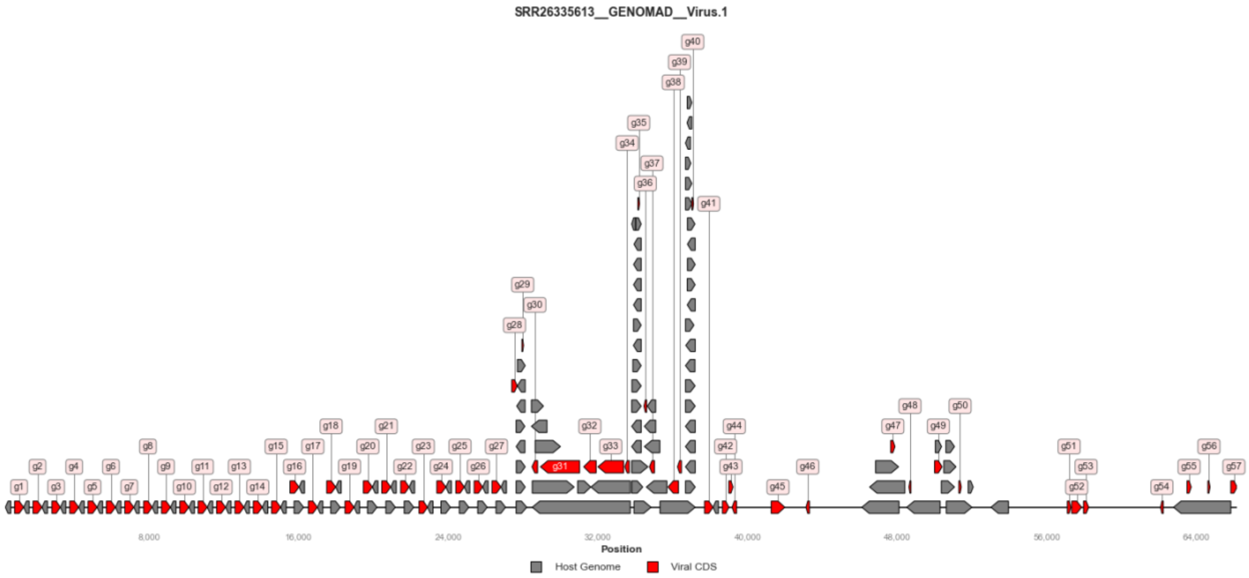

Figure S3 – Viral coding sequences and host homology of candidate integrated viral genome

Genomic neighborhood plot showing SRR26335613__GENOMAD__Virus.1. Gray indicates homology to host genome and red indicates viral CDS.

Figure S4 - Intergenic repeated components across multiple viruses in VSLC-1
IGRCs detected with 100% sequence identity in viral genomes.

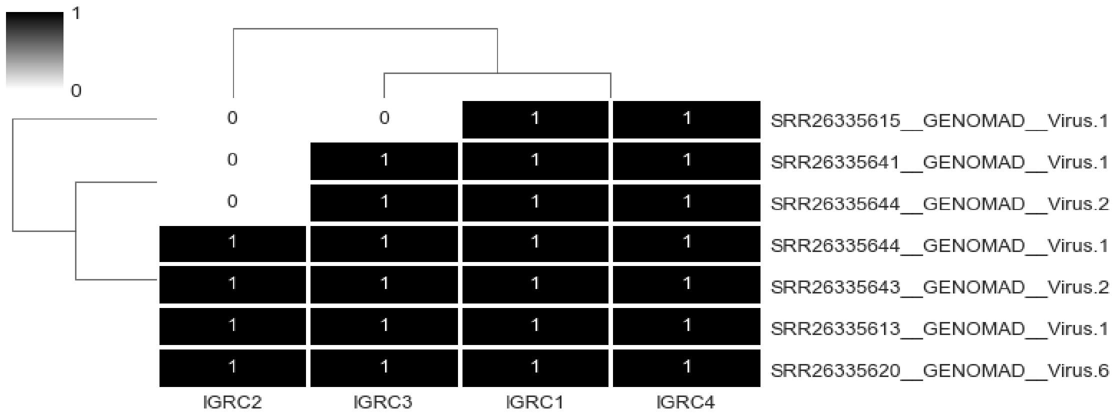

A.

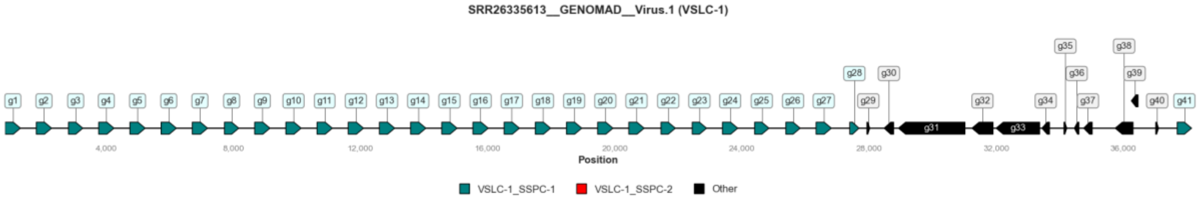
SRR26335613__GENOMAD__Virus.1 (VSLC-1)
4,000
8,000
12,000
16,000
20,000
24,000
28,000
32,000
36,000
Position
VSLC-1_SSPC-1
VSLC-1_SSPC-2
Other

B.

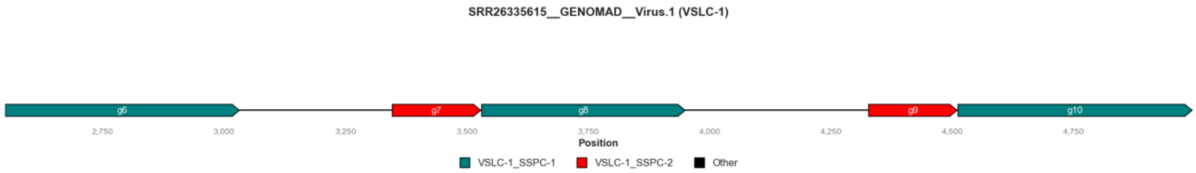
SRR26335615__GENOMAD__Virus.1 (VSLC-1)
2,750
3,000
3,250
3,500
3,750
4,000
4,250
4,500
4,750
Position
VSLC-1_SSPC-1
VSLC-1_SSPC-2
Other

C.

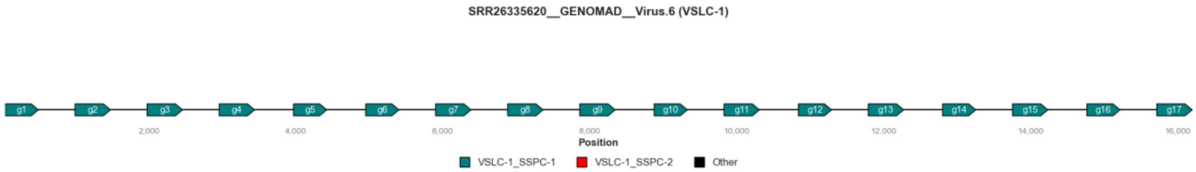
SRR26335620__GENOMAD__Virus.6 (VSLC-1)
2,000
4,000
6,000
8,000
10,000
12,000
14,000
16,000
Position
VSLC-1_SSPC-1
VSLC-1_SSPC-2
Other

D.

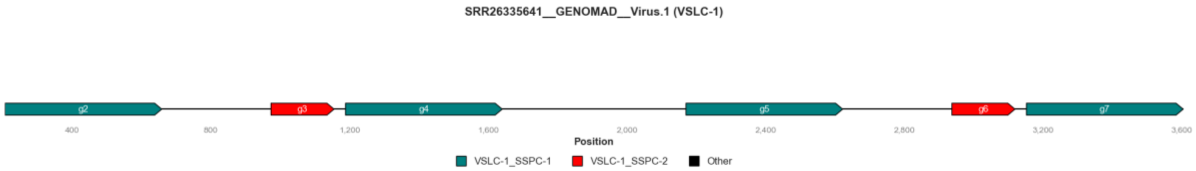
SRR26335641__GENOMAD__Virus.1 (VSLC-1)
400
800
1,200
1,600
2,000
2,400
2,800
3,200
3,600
Position
VSLC-1_SSPC-1
VSLC-1_SSPC-2
Other

E.

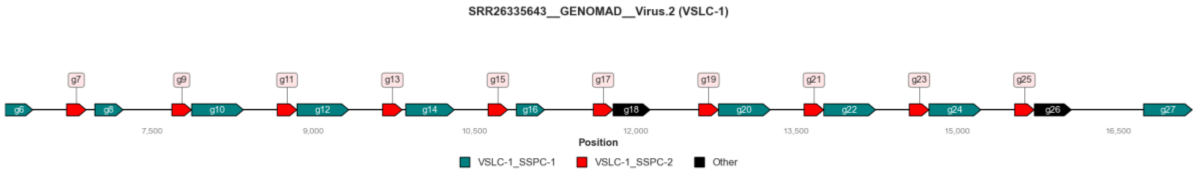
SRR26335643__GENOMAD__Virus.2 (VSLC-1)
7,500
9,000
10,500
12,000
13,500
15,000
16,500
Position
VSLC-1_SSPC-1
VSLC-1_SSPC-2
Other

F.

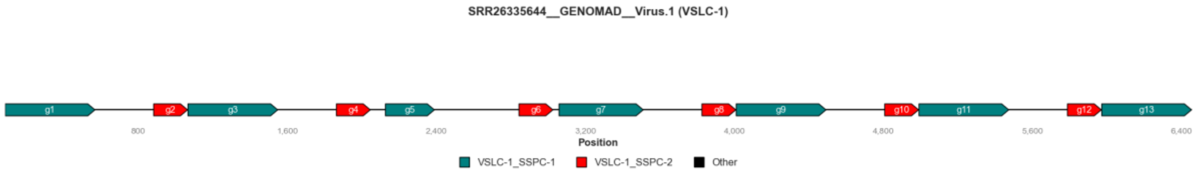
SRR26335644__GENOMAD__Virus.1 (VSLC-1)
800
1,600
2,400
3,200
4,000
4,800
5,600
6,400
Position
VSLC-1_SSPC-1
VSLC-1_SSPC-2
Other

G.

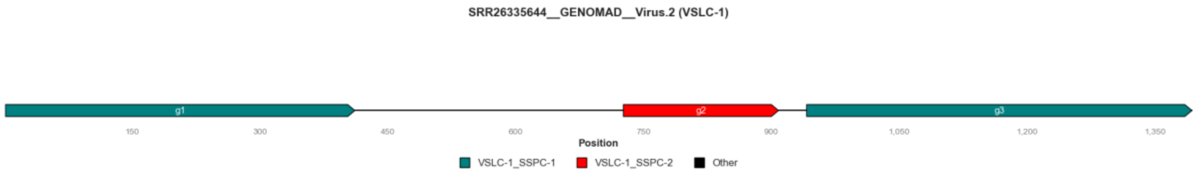

Figure S5 - Repeat paralogs across multiple viruses in VSLC-1

Genomic neighborhood plot for viral genomes that contain IGRCs with emphasis on repeat paralogs.

Supplementary Tables

Table S1 – Software dependencies, prevalence across modules, and licenses
Table S2 – Summary statistics of genomes and taxonomic classifications
Table S3 – Annotations for all protein-coding genes
Table S4 – BGCs for case study 2
Table S5 – Viral proteins from case study 3 homologous to known white-tailed deer retroviral proteins

Supplementary Appendix

Contains sequences for SRR26335613__GENOMAD__Virus.1 (VSLC-1): i) repeat paralog multiple sequence alignments (MSA); ii) intergenic sequences between repeat paralogs; iii) intergenic repeated components; and iv) intergenic repeated components (MSA).

i. SRR26335613__GENOMAD__Virus.1 (VSLC-1) repeat paralog (MSA)

>g19
MFQGAHGIALHAVQGNRASSRLEGKSHGFSRVAAGTWGTFSTYGGDGPSTLVFVQRSQDSCRVTRDTSGVSSRLGRAKGTLLEVS
RETQGPFPVATGILGFLSMFKRSQAPSNFEAVSSACLSGCQRHVRHSVERSRGPRAFSRDSTGGADIPSPCEMKD*
>g28
MV------FLELRRGPGVH----------SRLTAGM--------ALQPSCLFNEVRTPVEL---
RGTPQESLRGLAGQGTLLEVSRETQGPFPVATGISGM-SCY--ASLPAN-EPVTGAI---------------------------AEIPEK-----*
>g22
MQ--CRGIGPHL--------
AWRGKSHGFSRVAAGTWGTFSTYGGDGPSTLVFVQRSQDSCRVTRDTSGVSSRLGRAKGTLLEVSRETQGPFPVATGILGFLSMFK
RSQAPSNFEAVSSACLSRCQRHVRHSVERSRGPRAFSRDSTGGADIPSPCEMKD*
>g26
MQ--CRGIGPHL--------
AWRGKSHGFSRVAAGTWGTFSTYGGDGPSTLVFVQRSQDSCRVTRDTSGVSSRLGRAKGTLLEVSRETQGPFPVATGILGFLSMFK
RSQAPSNFEAVSSACLSRCQRHVRHSVERSRGPRAFSRDSTGGADIPSPCEMKD*
>g8
MQ--CRGIGPHL--------
AWRGKSHGFSRVAAGTWGTFSTYGGDGPSTLVFVQRSQDSCRVTRDTSGVSSRLGRAKGTLLEVSRETQGPFPVATGILGFLSMFK
RSQAPSNFEAVSSACLSRCQRHVRHSVERSRGPRAFSRDSTGGADIPSPCEMKD*
>g25
MQ--CRGIGPHL--------
AWRGKSHGFSRVAAGTWGTFSTYGGDGPSTLVFVQRSQDSCRVTRDTSGVSSRLGRAKGTLLEVSRETQGPFPVATGILGFLSMFK
RSQAPSNFEAVSSACLSRCQRHVRHSVERSRGPRAFSRDSTGGADIPSPCEMKD*
>g23
MQ--CRGIGPHL--------
AWRGKSHGFSRVAAGTWGTFSTYGGDGPSTLVFVQRSQDSCRVTRDTSGVSSRLGRAKGTLLEVSRETQGPFPVATGILGFLSMFK
RSQAPSNFEAVSSACLSRCQRHVRHSVERSRGPRAFSRDSTGGADIPSPCEMKD*
>g41

MQ--CRGIGPHL--------
AWRGKSHGFSRVAAGTWGTFSTYGGDGPSTLVFVQRSQDSCRVTRDTSGVSSRLGRAKGTLLEVSRETQGPFPVATGILGFLSMFK
RSQAPSNFEAVSSACLSRCQRHVRHSVERSRGPRAFSRDSTGGADIPSPCEMKD*
>g3
MQ--CRGIGPHL--------
AWRGKSHGFSRVAAGTWGTFSTYGGDGPSTLVFVQRSQDSCRVTRDTSGVSSRLGRAKGTLLEVSRETQGPFPVATGILGFLSMFK
RSQAPSNFEAVSSACLSRCQRHVRHSVERSRGPRAFSRDSTGGADIPSPCEMKD*
>g11
MFQGAHGIALHAVQGNRASSRLEGKSHGFSRVAAGTWGTFSTYGGDGPSTLVFVQRSQDSCRVTRDTSGVSSRLGRAKGTLLEVS
RETQGPFPVATGILGFLSMFKRSQAPSNFEAVSSACLSRCQRHVRHSVERSRGPRAFSRDSTGGADIPSPCEMKD*
>g21
MFQGAHGIALHAVQGNRASSRLEGKSHGFSRVAAGTWGTFSTYGGDGPSTLVFVQRSQDSCRVTRDTSGVSSRLGRAKGTLLEVS
RETQGPFPVATGILGFLSMFKRSQAPSNFEAVSSACLSRCQRHVRHSVERSRGPRAFSRDSTGGADIPSPCEMKD*
>g12
MFQGAHGIALHAVQGNRASSRLEGKSHGFSRVAAGTWGTFSTYGGDGPSTLVFVQRSQDSCRVTRDTSGVSSRLGRAKGTLLEVS
RETQGPFPVATGILGFLSMFKRSQAPSNFEAVSSACLSRCQRHVRHSVERSRGPRAFSRDSTGGADIPSPCEMKD*
>g7
MFQGAHGIALHAVQGNRASSRLEGKSHGFSRVAAGTWGTFSTYGGDGPSTLVFVQRSQDSCRVTRDTSGVSSRLGRAKGTLLEVS
RETQGPFPVATGILGFLSMFKRSQAPSNFEAVSSACLSRCQRHVRHSVERSRGPRAFSRDSTGGADIPSPCEMKD*
>g27
MFQGAHGIALHAVQGNRASSRLEGKSHGFSRVAAGTWGTFSTYGGDGPSTLVFVQRSQDSCRVTRDTSGVSSRLGRAKGTLLEVS
RETQGPFPVATGILGFLSMFKRSQAPSNFEAVSSACLSRCQRHVRHSVERSRGPRAFSRDSTGGADIPSPCEMKD*
>g5
MFQGAHGIALHAVQGNRASSRLEGKSHGFSRVAAGTWGTFSTYGGDGPSTLVFVQRSQDSCRVTRDTSGVSSRLGRAKGTLLEVS
RETQGPFPVATGILGFLSMFKRSQAPSNFEAVSSACLSRCQRHVRHSVERSRGPRAFSRDSTGGADIPSPCEMKD*
>g24
MFQGAHGIALHAVQGNRASSRLEGKSHGFSRVAAGTWGTFSTYGGDGPSTLVFVQRSQDSCRVTRDTSGVSSRLGRAKGTLLEVS
RETQGPFPVATGILGFLSMFKRSQAPSNFEAVSSACLSRCQRHVRHSVERSRGPRAFSRDSTGGADIPSPCEMKD*
>g1
MFQGAHGIALHAVQGNRASSRLEGKSHGFSRVAAGTWGTFSTYGGDGPSTLVFVQRSQDSCRVTRDTSGVSSRLGRAKGTLLEVS
RETQGPFPVATGILGFLSMFKRSQAPSNFEAVSSACLSRCQRHVRHSVERSRGPRAFSRDSTGGADIPSPCEMKD*
>g6
MFQGAHGIALHAVQGNRASSRLEGKSHGFSRVAAGTWGTFSTYGGDGPSTLVFVQRSQDSCRVTRDTSGVSSRLGRAKGTLLEVS
RETQGPFPVATGILGFLSMFKRSQAPSNFEAVSSACLSRCQRHVRHSVERSRGPRAFSRDSTGGADIPSPCEMKD*
>g18
MFQGAHGIALHAVQGNRASSRLEGKSHGFSRVAAGTWGTFSTYGGDGPSTLVFVQRSQDSCRVTRDTSGVSSRLGRAKGTLLEVS
RETQGPFPVATGILGFLSMFKRSQAPSNFEAVSSACLSRCQRHVRHSVERSRGPRAFSRDSTGGADIPSPCEMKD*
>g15
MFQGAHGIALHAVQGNRASSRLEGKSHGFSRVAAGTWGTFSTYGGDGPSTLVFVQRSQDSCRVTRDTSGVSSRLGRAKGTLLEVS
RETQGPFPVATGILGFLSMFKRSQAPSNFEAVSSACLSRCQRHVRHSVERSRGPRAFSRDSTGGADIPSPCEMKD*
>g4
MFQGAHGIALHAVQGNRASSRLEGKSHGFSRVAAGTWGTFSTYGGDGPSTLVFVQRSQDSCRVTRDTSGVSSRLGRAKGTLLEVS
RETQGPFPVATGILGFLSMFKRSQAPSNFEAVSSACLSRCQRHVRHSVERSRGPRAFSRDSTGGADIPSPCEMKD*
>g17
MFQGAHGIALHAVQGNRASSRLEGKSHGFSRVAAGTWGTFSTYGGDGPSTLVFVQRSQDSCRVTRDTSGVSSRLGRAKGTLLEVS
RETQGPFPVATGILGFLSMFKRSQAPSNFEAVSSACLSRCQRHVRHSVERSRGPRAFSRDSTGGADIPSPCEMKD*
>g10
MFQGAHGIALHAVQGNRASSRLEGKSHGFSRVAAGTWGTFSTYGGDGPSTLVFVQRSQDSCRVTRDTSGVSSRLGRAKGTLLEVS
RETQGPFPVATGILGFLSMFKRSQAPSNFEAVSSACLSRCQRHVRHSVERSRGPRAFSRDSTGGADIPSPCEMKD*
>g2
MFQGAHGIALHAVQGNRASSRLEGKSHGFSRVAAGTWGTFSTYGGDGPSTLVFVQRSQDSCRVTRDTSGVSSRLGRAKGTLLEVS
RETQGPFPVATGILGFLSMFKRSQAPSNFEAVSSACLSRCQRHVRHSVERSRGPRAFSRDSTGGADIPSPCEMKD*
>g14
MFQGAHGIALHAVQGNRASSRLEGKSHGFSRVAAGTWGTFSTYGGDGPSTLVFVQRSQDSCRVTRDTSGVSSRLGRAKGTLLEVS
RETQGPFPVATGILGFLSMFKRSQAPSNFEAVSSACLSRCQRHVRHSVERSRGPRAFSRDSTGGADIPSPCEMKD*
>g13
MFQGAHGIALHAVQGNRASSRLEGKSHGFSRVAAGTWGTFSTYGGDGPSTLVFVQRSQDSCRVTRDTSGVSSRLGRAKGTLLEVS
RETQGPFPVATGILGFLSMFKRSQAPSNFEAVSSACLSRCQRHVRHSVERSRGPRAFSRDSTGGADIPSPCEMKD*
>g20
MFQGAHGIALHAVQGNRASSRLEGKSHGFSRVAAGTWGTFSTYGGDGPSTLVFVQRSQDSCRVTRDTSGVSSRLGRAKGTLLEVS
RETQGPFPVATGILGFLSMFKRSQAPSNFEAVSSACLSRCQRHVRHSVERSRGPRAFSRDSTGGADIPSPCEMKD*
>g16
MFQGAHGIALHAVQGNRASSRLEGKSHGFSRVAAGTWGTFSTYGGDGPSTLVFVQRSQDSCRVTRDTSGVSSRLGRAKGTLLEVS
RETQGPFPVATGILGFLSMFKRSQAPSNFEAVSSACLSRCQRHVRHSVERSRGPRAFSRDSTGGADIPSPCEMKD*
>g9
MFQGAHGIALHAVQGNRASSRLEGKSHGFSRVAAGTWGTFSTYGGDGPSTLVFVQRSQDSCRVTRDTSGVSSRLGRAKGTLLEVS
RETQGPFPVATGILGFLSMFKRSQAPSNFEAVSSACLSRCQRHVRHSVERSRGPRAFSRDSTGGADIPSPCEMKD*

ii. SRR26335613__GENOMAD__Virus.1 (VSLC-1) intergenic sequences between repeat paralogs

>g1-g2 SRR26335613__contig_1497 SRR26335613__GENOMAD__Virus.1_1305:1803
ACTGTATTCAAGCCACTGCAAGGAAACCCGGCCTCCTTTCGAGTCAGGGCTTCTCGGTGTATATGCCCATTGAGGCAGCAATCTCAGGGTCCCTCCCTCATACCTATT
GCTGAGAGAAGCCTCCTCTTGAGGGGCTTGTGGAAAGGTGGCCTACGTCTTGAGTCTAAGCCAGGGAATCAGCTCTCATCTCGAGGCAATTTGGGGTACACGGAG
CAGTCCTCGAGTTGCTCTGCTGAACTTGGTACTCCTCTAGACTTGGCCCGTTGTTCTCGGGGAACCTCTGGAGTTGCCTAAAGGGAGTCAAGCCTCAGGTCGTGTT
TGATGGGGAACGGGGGATGGCTCTGGAGTCAATGCAGGGGAAGCGGGCCTCATCTCGAGTTGATTTGGGGCCCATGGAGCTCCTTCGCGATGCTGGTGTGACCT
CAGGGTCCCTCTCTACTTGTGACAGTGTTTTTGCGGACTGTCTCGACATCCATCAAGCAAGTCGAGGCTCCTGAC
>g2-g3 SRR26335613__contig_1497 SRR26335613__GENOMAD__Virus.1_2286:2815
ACTGTATTCAAGCCACTGCAAGGAAACCCGGCCTCCTTTCGAGTCAGGGCTTCTCGGTGTATATGCCCATTGAGGCAGCAATCTCAGGGTCCCTCCCTCATACCTATT
GCTGAGAGAAGCCTCCTCTTGAGGGGCTTGTGGAAAGGTGGCCTACGTCTTGAGTCTAAGCCAGGGAATCAGCTCTCATCTCGAGGCAATTTGGGGTACACGGAG
CAGTCCTCGAGTTGCTCTGCTGAACTTGGTACTCCTCTAGACTTGGCCCGTTGTTCTCGGGGAACCTCTGGAGTTGCCTAAAGGGAGTCAAGCCTCAGGTCGTGTT
TGATGGGGAACGGGGGATGGCTCTGGAGTCAATGCAGGGGAAGCGGGCCTCATCTCGAGTTGATTTGGGGCCCATGGAGCTCCTTCGCGATGCTGGTGTGACCT
CAGGGTCCCTCTCTACTTGTGACAGTGTTTTTGCGGACTGTCTCGACATCCATCAAGCAAGTCGAGGCTCCTGACATGTTTCAGGGGGCTCACGGAATTGCTCTGC
>g3-g4 SRR26335613__contig_1497 SRR26335613__GENOMAD__Virus.1_3268:3766
ACTGTATTCAAGCCACTGCAAGGAAACCCGGCCTCCTTTCGAGTCAGGGCTTCTCGGTGTATATGCCCATTGAGGCAGCAATCTCAGGGTCCCTCCCTCATACCTATT
GCTGAGAGAAGCCTCCTCTTGAGGGGCTTGTGGAAAGGTGGCCTACGTCTTGAGTCTAAGCCAGGGAATCAGCTCTCATCTCGAGGCAATTTGGGGTACACGGAG
CAGTCCTCGAGTTGCTCTGCTGAACTTGGTACTCCTCTAGACTTGGCCCGTTGTTCTCGGGGAACCTCTGGAGTTGCCTAAAGGGAGTCAAGCCTCAGGTCGTGTT
TGATGGGGAACGGGGGATGGCTCTGGAGTCAATGCAGGGGAAGCGGGCCTCATCTCGAGTTGATTTGGGGCCCATGGAGCTCCTTCGCGATGCTGGTGTGACCT
CAGGGTCCCTCTCTACTTGTGACAGTGTTTTTGCGGACTGTCTCGACATCCATCAAGCAAGTCGAGGCTCCTGAC
>g4-g5 SRR26335613__contig_1497 SRR26335613__GENOMAD__Virus.1_4249:4747
ACTGTATTCAAGCCACTGCAAGGAAACCCGGCCTCCTTTCGAGTCAGGGCTTCTCGGTGTATATGCCCATTGAGGCAGCAATCTCAGGGTCCCTCCCTCATACCTATT
GCTGAGAGAAGCCTCCTCTTGAGGGGCTTGTGGAAAGGTGGCCTACGTCTTGAGTCTAAGCCAGGGAATCAGCTCTCATCTCGAGGCAATTTGGGGTACACGGAG
CAGTCCTCGAGTTGCTCTGCTGAACTTGGTACTCCTCTAGACTTGGCCCGTTGTTCTCCGGGAACCTCTGGAGTTGCCTAAAGGGAGTCAAGCCTCAGGTCGTGTTT
GATGGGGAACGGGGGATGGCTCTGGAGTCAATGCAGGGGAAGCGGGCCTCATCTCGAGTTGATTTGGGGCCCATGGAGCTCCTTCGCGATGCTGGTGTGACCTC
AGGGTCCCTCTCTACTTGTGACAGTGTTTTTGCGGACTGTCTCGACATCCATCAAGCAAGTCGAGGCTCCTGAC
>g5-g6 SRR26335613__contig_1497 SRR26335613__GENOMAD__Virus.1_5230:5728
ACTGTATTCAAGCCACTGCAAGGAAACCCGGCCTCCTTTCGAGTCAGGGCTTCTCGGTGTATATGCCCATTGAGGCAGCAATCTCAGGGTCCCTCCCTCATACCTATT
GCTGAGAGAAGCCTCCTCTTGAGGGGCTTGTGGAAAGGTGGCCTACGTCTTGAGTCTAAGCCAGGGAATCAGCTCTCATCTCGAGGCAATTTGGGGTACACGGAG
CAGTCCTCGAGTTGCTCTGCTGAACTTGGTACTCCTCTAGACTTGGCCCGTTGTTCTCCGGGAACCTCTGGAGTTGCCTAAAGGGAGTCAAGCCTCAGGTCGTGTTT
GATGGGGAACGGGGGATGGCTCTGGAGTCAATGCAGGGGAAGCGGGCCTCATCTCGAGTTGATTTGGGGCCCATGGAGCTCCTTCGCGATGCTGGTGTGACCTC
AGGGTCCCTCTCTACTTGTGACAGTGTTTTTGCGGACTGTCTCGACATCCATCAAGCAAGTCGAGGCTCCTGAC
>g6-g7 SRR26335613__contig_1497 SRR26335613__GENOMAD__Virus.1_6211:6709
ACTGTATTCAAGCCACTGCAAGGAAACCCGGCCTCCTTTCGAGTCAGGGCTTCTCGGTGTATATGCCCATTGAGGCAGCAATCTCAGGGTCCCTCCCTCATACCTATT
GCTGAGAGAAGCCTCCTCTTGAGGGGCTTGTGGAAAGGTGGCCTACGTCTTGAGTCTAAGCCAGGGAATCAGCTCTCATCTCGAGGCAATTTGGGGTACACGGAG
CAGTCCTCGAGTTGCTCTGCTGAACTTGGTACTCCTCTAGACTTGGCCCGTTGTTCTCGGGGAACCTCTGGAGTTGCCTAAAGGGAGTCAAGCCTCAGGTCGTGTT
TGATGGGGAACGGGGGATGGCTCTGGAGTCAATGCAGGGGAAGCGGGCCTCATCTCGAGTTGATTTGGGGCCCATGGAGCTCCTTCGCGATGCTGGTGTGACCT
CAGGGTCCCTCTCTACTTGTGACAGTGTTTTTGCGGACTGTCTCGACATCCATCAAGCAAGTCGAGGCTCCTGAC
>g7-g8 SRR26335613__contig_1497 SRR26335613__GENOMAD__Virus.1_7192:7721
ACTGTATTCAAGCCACTGCAAGGAAACCCGGCCTCCTTTCGAGTCAGGGCTTCTCGGTGTATATGCCCATTGAGGCAGCAATCTCAGGGTCCCTCCCTCATACCTATT
GCTGAGAGAAGCCTCCTCTTGAGGGGCTTGTGGAAAGGTGGCCTACGTCTTGAGTCTAAGCCAGGGAATCAGCTCTCATCTCGAGGCAATTTGGGGTACACGGAG
CAGTCCTCGAGTTGCTCTGCTGAACTTGGTACTCCTCTAGACTTGGCCCGTTGTTCTCGGGGAACCTCTGGAGTTGCCTAAAGGGAGTCAAGCCTCAGGTCGTGTT
TGATGGGGAACGGGGGATGGCTCTGGAGTCAATGCAGGGGAAGCGGGCCTCATCTCGAGTTGATTTGGGGCCCATGGAGCTCCTTCGCGATGCTGGTGTGACCT
CAGGGTCCCTCTCTACTTGTGACAGTGTTTTTGCGGACTGTCTCGACATCCATCAAGCAAGTCGAGGCTCCTGACATGTTTCAGGGGGCTCACGGAATTGCTCTGC
>g8-g9 SRR26335613__contig_1497 SRR26335613__GENOMAD__Virus.1_8174:8672
ACTGTATTCAAGCCACTGCAAGGAAACCCGGCCTCCTTTCGAGTCAGGGCTTCTCGGTGTATATGCCCATTGAGGCAGCAATCTCAGGGTCCCTCCCTCATACCTATT
GCTGAGAGAAGCCTCCTCTTGAGGGGCTTGTGGAAAGGTGGCCTACGTCTTGAGTCTAAGCCAGGGAATCAGCTCTCATCTCGAGGCAATTTGGGGTACACGGAG
CAGTCCTCGAGTTGCTCTGCTGAACTTGGTACTCGTCTAGACTTGGCCCGTTGTTCTCCGGGAACCTCTGGAGTTGCCTAAAGGGAGTCAAGCCTCAGGTCGTGTT
TGATGGGGAACTGGGGATGGCTCTGGAGTCAATGCAGGGGAAGCGGGCCTCATCTCGAGTTGATTTGGGGCCCATGGAGCTCCTTCGCGATGCTGGTGTGACCTC
AGGGTCCCTCTCTACTTGTGACAGTGTTTTTGCGGACTGTCTCGACATCCATCAAGCAAGTCGAGGCTCCTGAC
>g9-g10 SRR26335613__contig_1497 SRR26335613__GENOMAD__Virus.1_9155:9653
ACTGTATTCAAGCCACTGCAAGGAAACCCGGCCTCCTTTCGAGTCAGGGCTTCTCGGTGTATATGCCCATTGAGGCAGCAATCTCAGGGTCCCTCCCTCATACCTATT
GCTGAGAGAAGCCTCCTCTTGAGGGGCTTGTGGAAAGGTGGCCTACGTCTTGAGTCTAAGCCAGGGAATCAGCTCTCATCTCGAGGCAATTTGGGGTACACGGAG
CAGTCCTCGAGTTGCTCTGCTGAACTTGGTACTCCTCTAGACTTGGCCCGTTGTTCTCGGGGAACCTCTGGAGTTGCCTAAAGGGAGTCAAGCCTCAGGTCGTGTT
TGATGGGGAACGGGGGATGGCTCTGGAGTCAATGCAGGGGAAGCGGGCCTCATCTCGAGTTGATTTGGGGCCCATGGAGCTCCTTCGCGATGCTGGTGTGACCT
CAGGGTCCCTCTCTACTTGTGACAGTGTTTTTGCGGACTGTCTCGACATCCATCAAGCAAGTCGAGGCTCCTGAC
>g10-g11 SRR26335613__contig_1497 SRR26335613__GENOMAD__Virus.1_10136:10634
ACTGTATTCAAGCCACTGCAAGGAAACCCGGCCTCCTTTCGAGTCAGGGCTTCTCGGTGTATATGCCCATTGAGGCAGCAATCTCAGGGTCCCTCCCTCATACCTATT
GCTGAGAGAAGCCTCCTCTTGAGGGGCTTGTGGAAAGGTGGCCTACGTCTTGAGTCTAAGCCAGGGAATCAGCTCTCATCTCGAGGCAATTTGGGGTACACGGAG
CAGTCCTCGAGTTGCTCTGCTGAACTTGGTACTCCTCTAGACTTGGCCCGTTGTTCTCCGGGAACCTCTGGAGTTGCCTAAAGGGAGTCAAGCCTCAGGTCGTGTTT
GATGGGGAACAGGGGATGGCTCTGGAGTCAATGCAGGGGAAGCGGGCCTCATCTCGAGTTGATTTGGGGCCCATGGAGCTCCTTCGCGATGCTGGTGTGACCTC
AGGGTCCCTCTCTACTTGTGACAGTGTTTTTGCGGACTGTCTCGACATCCATCAAGCAAGTCGAGGCTCCTGAC
>g11-g12 SRR26335613__contig_1497 SRR26335613__GENOMAD__Virus.1_11117:11615
ACTGTATTCAAGCCACTGCAAGGAAACCCGGCCTCCTTTCGAGTCAGGGCTTCTCGGTGTATATGCCCATTGAGGCAGCAATCTCAGGGTCCCTCCCTCATACCTATT
GCTGAGAGAAGCCTCCTCTTGAGGGGCTTGTGGAAAGGTGGCCTACGTCTTGAGTCTAAGCCAGGGAATCAGCTCTCATCTCGAGGCAATTTGGGGTACACGGAG
CAGTCCTCGAGTTGCTCTGCTGAACTTGGTACTCCTCTAGACTTGGCCCGTTGTTCTCGGGGAACCTCTGGAGTTGCCTAAAGGGAGTCAAGCCTCAGGTCGTGTT
TGATGGGGAACGGGGGATGGCTCTGGAGTCAATGCAGGGGAAGCGGGCCTCATCTCGAGTTGATTTGGGGCCCATGGAGCTCCTTCGCGATGCTGGTGTGACCT
CAGGGTCCCTCTCTACTTGTGACAGTGTTTTTGCGGACTGTCTCGACATCCATCAAGCAAGTCGAGGCTCCTGAC

>g12-g13 SRR26335613__contig_1497 SRR26335613__GENOMAD__Virus.1_12098:12596
ACTGTATTCAAGCCACTGCAAGGAAACCCGGCCTCCTTTCGAGTCAGGGCTTCTCGGTGTATATGCCCATTGAGGCAGCAATCTCAGGGTCCCTCCCTCATACCTATT
GCTGAGAGAAGCCTCCTCTTGAGGGGCTTGTGGAAAGGTGGCCTACGTCTTGAGTCTAAGCCAGGGAATCAGCTCTCATCTCGAGGCAATTTGGGGTACACGGAG
CAGTCCTCGAGTTGCTCTGCTGAACTTGGTACTCCTCTAGACTTGGCCCGTTGTTCTCGGGGAACCTCTGGAGTTGCCTAAAGGGAGTCAAGCCTCAGGTCGTGTT
TGATGGGGAACGGGGGATGGCTCTGGAGTCAATGCAGGGGAAGCGGGCCTCATCTCGAGTTGATTTGGGGCCCATGGAGCTCCTTCGCGATGCTGGTGTGACCT
CAGGGTCCCTCTCTACTTGTGACAGTGTTTTTGCGGACTGTCTCGACATCCATCAAGCAAGTCGAGGCTCCTGAC
>g13-g14 SRR26335613__contig_1497 SRR26335613__GENOMAD__Virus.1_13079:13577
ACTGTATTCAAGCCACTGCAAGGAAACCCGGCCTCCTTTCGAGTCAGGGCTTCTCGGTGTATATGCCCATTGAGGCAGCAATCTCAGGGTCCCTCCCTCATACCTATT
GCTGAGAGAAGCCTCCTCTTGAGGGGCTTGTGGAAAGGTGGCCTACGTCTTGAGTCTAAGCCAGGGAATCAGCTCTCATCTCGAGGCAATTTGGGGTACACGGAG
CAGTCCTCGAGTTGCTCTGCTGAACTTGGTACTCCTCTAGACTTGGCCCGTTGTTCTCGGGGAACCTCTGGAGTTGCCTAAAGGGAGTCAAGCCTCAGGTCGTGTT
TGATGGGGAACGGGGGATGGCTCTGGAGTCAATGCAGGGGAAGCGGGCCTCATCTCGAGTTGATTTGGGGCCCATGGAGCTCCTTCGCGATGCTGGTGTGACCT
CAGGGTCCCTCTCTACTTGTGACAGTGTTTTTGCGGACTGTCTCGACATCCATCAAGCAAGTCGAGGCTCCTGAC
>g14-g15 SRR26335613__contig_1497 SRR26335613__GENOMAD__Virus.1_14060:14558
ACTGTATTCAAGCCACTGCAAGGAAACCCGGCCTCCTTTCGAGTCAGGGCTTCTCGGTGTATATGCCCATTGAGGCAGCAATCTCAGGGTCCCTCCCTCATACCTATT
GCTGAGAGAAGCCTCCTCTTGAGGGGCTTGTGGAAAGGTGGCCTACGTCTTGAGTCTAAGCCAGGGAATCAGCTCTCATCTCGAGGCAATTTGGGGTACACGGAG
CAGTCCTCGAGTTGCTCTGCTGAACTTGGTACTCCTCTAGACTTGGCCCGTTGTTCTCGGGGAACCTCTGGAGTTGCCTAAAGGGAGTCAAGCCTCAGGTCGTGTT
TGATGGGGAACGGGGGATGGCTCTGGAGTCAATGCAGGGGAAGCGGGCCTCATCTCGAGTTGATTTGGGGCCCATGGAGCTCCTTCGCGATGCTGGTGTGACCT
CAGGGTCCCTCTCTACTTGTGACAGTGTTTTTGCGGACTGTCTCGACATCCATCAAGCAAGTCGAGGCTCCTGAC
>g15-g16 SRR26335613__contig_1497 SRR26335613__GENOMAD__Virus.1_15041:15539
ACTGTATTCAAGCCACTGCAAGGAAACCCGGCCTCCTTTCGAGTCAGGGCTTCTCGGTGTATATGCCCATTGAGGCAGCAATCTCAGGGTCCCTCCCTCATACCTATT
GCTGAGAGAAGCCTCCTCTTGAGGGGCTTGTGGAAAGGTGGCCTACGTCTTGAGTCTAAGCCAGGGAATCAGCTCTCATCTCGAGGCAATTTGGGGTACACGGAG
CAGTCCTCGAGTTGCTCTGCTGAACTTGGTACTCCTCTAGACTTGGCCCGTTGTTCTCGGGGAACCTCTGGAGTTGCCTAAAGGGAGTCAAGCCTCAGGTCGTGTT
TGATGGGGAACGGGGGATGGCTCTGGAGTCAATGCAGGGGAAGCGGGCCTCATCTCGAGTTGATTTGGGGCCCATGGAGCTCCTTCGCGATGCTGGTGTGACCT
CAGGGTCCCTCTCTACTTGTGACAGTGTTTTTGCGGACTGTCTCGACATCCATCAAGCAAGTCGAGGCTCCTGAC
>g16-g17 SRR26335613__contig_1497 SRR26335613__GENOMAD__Virus.1_16022:16520
ACTGTATTCAAGCCACTGCAAGGAAACCCGGCCTCCTTTCGAGTCAGGGCTTCTCGGTGTATATGCCCATTGAGGCAGCAATCTCAGGGTCCCTCCCTCATACCTATT
GCTGAGAGAAGCCTCCTCTTGAGGGGCTTGTGGAAAGGTGGCCTACGTCTTGAGTCTAAGCCAGGGAATCAGCTCTCATCTCGAGGCAATTTGGGGTACACGGAG
CAGTCCTCGAGTTGCTCTGCTGAACTTGGTACTCCTCTAGACTTGGCCCGTTGTTCTCGGGGAACCTCTGGAGTTGCCTAAAGGGAGTCAAGCCTCAGGTCGTGTT
TGATGGGGAACGGGGGATGGCTCTGGAGTCAATGCAGGGGAAGCGGGCCTCATCTCGAGTTGATTTGGGGCCCATGGAGCTCCTTCGCGATGCTGGTGTGACCT
CAGGGTCCCTCTCTACTTGTGACAGTGTTTTTGCGGACTGTCTCGACATCCATCAAGCAAGTCGAGGCTCCTGAC
>g17-g18 SRR26335613__contig_1497 SRR26335613__GENOMAD__Virus.1_17003:17500
ACTGTATTCAAGCCACTGCAAGGAAACCCGGCCTCCTTTCGAGTCAGGGCTTCTCGGTGTATATGCCCATTGAGGCAGCAATCTCAGGGTCCCTCCCTCATACCTATT
GCTGAGAGAAGCCTCCTCTTGAGGGGCTTGTGGAAAGGTGGCCTACGTCTTGAGTCTAAGCCAGGGAATCAGCTCTCATCTCGAGGCAATTTGGGGTACACGGAG
CAGTCCTCGAGTTGCTCTGCTGAACTTGGTACTCCTCTAGACTTGGCCCGTTGTTCTCGGGAACCTCTGGAGTTGCCTAAAGGGAGTCAAGCCTCAGGTCGTGTTT
GATGGGGAACGGGGGATGGCTCTGGAGTCAATGCAGGGGAAGCGGGCCTCATCTCGAGTTGATTTGGGGCCCATGGAGCTCCTTCGCGATGCTGGTGTGACCTC
AGGGTCCCTCTCTACTTGTGACAGTGTTTTTGCGGACTGTCTCGACATCCATCAAGCAAGTCGAGGCTCCTGAC
>g18-g19 SRR26335613__contig_1497 SRR26335613__GENOMAD__Virus.1_17983:18481
ACTGTATTCAAGCCACTGCAAGGAAACCCGGCCTCCTTTCGAGTCAGGGCTTCTCGGTGTATATGCCCATTGAGGCAGCAATCTCAGGGTCCCTCCCTCATACCTATT
GCTGAGAGAAGCCTCCTCTTGAGGGGCTTGTGGAAAGGTGGCCTACGTCTTGAGTCTAAGCCAGGGAATCAGCTCTCATCTCGAGGCAATTTGGGGTACACGGAG
CAGTCCTCGAGTTGCTCTGCTGAACTTGGTACTCCTCTAGACTTGGCCCGTTGTTCTCGGGGAACCTCTGGAGTTGCCTAAAGGGAGTCAAGCCTCAGGTCGTGTT
TGATGGGGAACGGGGGATGGCTCTGGAGTCAATGCAGGGGAAGCGGGCCTCATCTCGAGTTGATTTGGGGCCCATGGAGCTCCTTCGCGATGCTGGTGTGACCT
CAGGGTCCCTCTCTACTTGTGACAGTGTTTTTGCGGACTGTCTCGACATCCATCAAGCAAGTCGAGGCTCCTGAC
>g19-g20 SRR26335613__contig_1497 SRR26335613__GENOMAD__Virus.1_18964:19462
ACTGTATTCAAGCCACTGCAAGGAAACCCGGCCTCCTTTCGAGTCAGGGCTTCTCGGTGTATATGCCCATTGAGGCAGCAATCTCAGGGTCCCTCCCTCATACCTATT
GCTGAGAGAAGCCTCCTCTTGAGGGGCTTGTGGAAAGGTGGCCTACGTCTTGAGTCTAAGCCAGGGAATCAGCTCTCATCTCGAGGCAATTTGGGGTACACGGAG
CAGTCCTCGAGTTGCTCTGCTGAACTTGGTACTCCTCTAGACTTGGCCCGTTGTTCTCGGGGAACCTCTGGAGTTGCCTAAAGGGAGTCAAGCCTCAGGTCGTGTT
TGATGGGGAACGGGGGATGGCTCTGGAGTCAATGCAGGGGAAGCGGGCCTCATCTCGAGTTGATTTGGGGCCCATGGAGCTCCTTCGCGATGCTGGTGTGACCT
CAGGGTCCCTCTCTACTTGTGACAGTGTTTTTGCGGACTGTCTCGACATCCATCAAGCAAGTCGAGGCTCCTGAC
>g20-g21 SRR26335613__contig_1497 SRR26335613__GENOMAD__Virus.1_19945:20443
ACTGTATTCAAGCCACTGCAAGGAAACCCGGCCTCCTTTCGAGTCAGGGCTTCTCGGTGTATATGCCCATTGAGGCAGCAATCTCAGGGTCCCTCCCTCATACCTATT
GCTGAGAGAAGCCTCCTCTTGAGGGGCTTGTGGAAAGGTGGCCTACGTCTTGAGTCTAAGCCAGGGAATCAGCTCTCATCTCGAGGCAATTTGGGGTACACGGAG
CAGTCCTCGAGTTGCTCTGCTGAACTTGGTACTCCTCTAGACTTGGCCCGTTGTTCTCGGGGAACCTCTGGAGTTGCCTAAAGGGAGTCAAGCCTCAGGTCGTGTT
TGATGGGGAACGGGGGATGGCTCTGGAGTCAATGCAGGGGAAGCGGGCCTCATCTCGAGTTGATTTGGGGCCCATGGAGCTCCTTCGCGATGCTGGTGTGACCT
CAGGGTCCCTCTCTACTTGTGACAGTGTTTTTGCGGACTGTCTCGACATCCATCAAGCAAGTCGAGGCTCCTGAC
>g21-g22 SRR26335613__contig_1497 SRR26335613__GENOMAD__Virus.1_20926:21455
ACTGTATTCAAGCCACTGCAAGGAAACCCGGCCTCCTTTCGAGTCAGGGCTTCTCGGTGTATATGCCCATTGAGGCAGCAATCTCAGGGTCCCTCCCTCATACCTATT
GCTGAGAGAAGCCTCCTCTTGAGGGGCTTGTGGAAAGGTGGCCTACGTCTTGAGTCTAAGCCAGGGAATCAGCTCTCATCTCGAGGCAATTTGGGGTACACGGAG
CAGTCCTCGAGTTGCTCTGCTGAACTTGGTACTCCTCTAGACTTGGCCCGTTGTTCTCGGGGAACCTCTGGAGTTGCCTAAAGGGAGTCAAGCCTCAGGTCGTGTT
TGATGGGGAACGGGGGATGGCTCTGGAGTCAATGCAGGGGAAGCGGGCCTCATCTCGAGTTGATTTGGGGCCCATGGAGCTCCTTCGCGATGCTGGTGTGACCT
CAGGGTCCCTCTCTACTTGTGACAGTGTTTTTGCGGACTGTCTCGACATCCATCAAGCAAGTCGAGGCTCCTGACATGTTTCAGGGGGCTCACGGAATTGCTCTGC
>g22-g23 SRR26335613__contig_1497 SRR26335613__GENOMAD__Virus.1_21908:22437
ACTGTATTCAAGCCACTGCAAGGAAACCCGGCCTCCTTTCGAGTCAGGGCTTCTCGGTGTATATGCCCATTGAGGCAGCAATCTCAGGGTCCCTCCCTCATACCTATT
GCTGAGAGAAGCCTCCTCTTGAGGGGCTTGTGGAAAGGTGGCCTACGTCTTGAGTCTAAGCCAGGGAATCAGCTCTCATCTCGAGGCAATTTGGGGTACACGGAG
CAGTCCTCGAGTTGCTCTGCTGAACTTGGTACTCCTCTAGACTTGGCCCGTTGTTCTCGGGGAACCTCTGGAGTTGCCTAAAGGGAGTCAAGCCTCAGGTCGTGTT
TGATGGGGAACGGGGGATGGCTCTGGAGTCAATGCAGGGGAAGCGGGCCTCATCTCGAGTTGATTTGGGGCCCATGGAGCTCCTTCGCGATGCTGGTGTGACCT
CAGGGTCCCTCTCTACTTGTGACAGTGTTTTTGCGGACTGTCTCGACATCCATCAAGCAAGTCGAGGCTCCTGACATGTTTCAGGGGGCTCACGGAATTGCTCTGC

>g23-g24 SRR26335613__contig_1497 SRR26335613__GENOMAD__Virus.1_22890:23388
ACTGTATTCAAGCCACTGCAAGGAAACCCGGCCTCCTTTCGAGTCAGGGCTTCTCGGTGTATATGCCCATTGAGGCAGCAATCTCAGGGTCCCTCCCTCATACCTATT
GCTGAGAGAAGCCTCCTCTTGAGGGGCTTGTGGAAAGGTGGCCTACGTCTTGAGTCTAAGCCAGGGAATCAGCTCTCATCTCGAGGCAATTTGGGGTACACGGAG
CAGTCCTCGAGTTGCTCTGCTGAACTTGGTACTCCTCTAGACTTGGCCCGTTGTTCTCGGGGAACCTCTGGAGTTGCCTAAAGGGAGTCAAGCCTCAGGTCGTGTT
TGATGGGGAACGGGGGATGGCTCTGGAGTCAATGCAGGGGAAGCGGGCCTCATCTCGAGTTGATTTGGGGCCCATGGAGCTCCTTCGCGATGCTGGTGTGACCT
CAGGGTCCCTCTCTACTTGTGACAGTGTTTTTGCGGACTGTCTCGACATCCATCAAGCAAGTCGAGGCTCCTGAC
>g24-g25 SRR26335613__contig_1497 SRR26335613__GENOMAD__Virus.1_23871:24400
ACTGTATTCAAGCCACTGCAAGGAAACCCGGCCTCCTTTCGAGTCAGGGCTTCTCGGTGTATATGCCCATTGAGGCAGCAATCTCAGGGTCCCTCCCTCATACCTATT
GCTGAGAGAAGCCTCCTCTTGAGGGGCTTGTGGAAAGGTGGCCTACGTCTTGAGTCTAAGCCAGGGAATCAGCTCTCATCTCGAGGCAATTTGGGGTACACGGAG
CAGTCCTCGAGTTGCTCTGCTGAACTTGGTACTCCTCTAGACTTGGCCCGTTGTTCTCGGGGAACCTCTGGAGTTGCCTAAAGGGAGTCAAGCCTCAGGTCGTGTT
TGATGGGGAACGGGGGATGGCTCTGGAGTCAATGCAGGGGAAGCGGGCCTCATCTCGAGTTGATTTGGGGCCCATGGAGCTCCTTCGCGATGCTGGTGTGACCT
CAGGGTCCCTCTCTACTTGTGACAGTGTTTTTGCGGACTGTCTCGACATCCATCAAGCAAGTCGAGGCTCCTGACATGTTTCAGGGGGCTCACGGAATTGCTCTGC
>g25-g26 SRR26335613__contig_1497 SRR26335613__GENOMAD__Virus.1_24853:25382
ACTGTATTCAAGCCACTGCAAGGAAACCCGGCCTCCTTTCGAGTCAGGGCTTCTCGGTGTATATGCCCATTGAGGCAGCAATCTCAGGGTCCCTCCCTCATACCTATT
GCTGAGAGAAGCCTCCTCTTGAGGGGCTTGTGGAAAGGTGGCCTACGTCTTGAGTCTAAGCCAGGGAATCAGCTCTCATCTCGAGGCAATTTGGGGTACACGGAG
CAGTCCTCGAGTTGCTCTGCTGAACTTGGTACTCCTCTAGACTTGGCCCGTTGTTCTCCGGGAACCTCTGGAGTTGCCTAAAGGGAGTCAAGCCTCAGGTCGTGTTT
GATGGGGAACGGGGGATGGCTCTGGAGTCAATGCAGGGGAAGCGGGCCTCATCTCGAGTTGATTTGGGGCCCATGGAGCTCCTTCGCGATGCTGGTGTGACCTC
AGGGTCCCTCTCTACTTGTGACAGTGTTTTTGCGGACTGTCTCGACATCCATCAAGCAAGTCGAGGCTCCTGACATGTTTCAGGGGGCTCACGGAATTGCTCTGC
>g26-g27 SRR26335613__contig_1497 SRR26335613__GENOMAD__Virus.1_25835:26333
ACTGTATTCAAGCCACTGCAAGGAAACCCGGCCTCCTTTCGAGTCAGGGCTTCTCGGTGTATATGCCCATTGAGGCAGCAATCTCAGGGTCCCTCCCTCATACCTATT
GCTGAGAGAAGCCTCCTCTTGAGGGGCTTGTGGAAAGGTGGCCTACGTCTTGAGTCTAAGCCAGGGAATCAGCTCTCATCTCGAGGCAATTTGGGGTACACGGAG
CAGTCCTCGAGTTGCTCTGCTGAACTTGGTACTCCTCTAGACTTGGCCCGTTGTTCTCGGGGAACCTCTGGAGTTGCCTAAAGGGAGTCAAGCCTCAGGTCGTGTT
TGATGGGGAACGGGGGATGGCTCTGGAGTCAATGCAGGGGAAGCGGGCCTCATCTCGAGTTGATTTGGGGCCCATGGAGCTCCTTCGCGATGCTGGTGTGACCT
CAGGGTCCCTCTCTACTTGTGACAGTGTTTTTGCGGACTGTCTCGACATCCATCAAGCAAGTCGAGGCTCCTGAC
>g27-g28 SRR26335613__contig_1497 SRR26335613__GENOMAD__Virus.1_26816:27393
ACTGTATTCAAGCCACTGCAAGGAAACCCGGCCTCCTTTCGAGTCAGGGCTTCTCGGTGTATATGCCCATTGAGGCAGCAATCTCAGGGTCCCTCCCTCATACCTATT
GCTGAGAGAAGCCTCCTCTTGAGGGGCTTGTGGAAAGGTGGCCTACGTCTTGAGTCTAAGCCAGGGAATCAGCTCTCATCTCGAGGCAATTTGGGGTACACGGAG
CAGTCCTCGAGTTGCTCTGCTGAACTTGGTACTCCTCTAGACTTGGCCCGTTGTTCTCGGGGAACCTCTGGAGTTGCCTAAAGGGAGTCAAGCCTCAGGTCGTGTT
TGATGGGGAACGGGGGATGGCTCTGGAGTCAATGCAGGGGAAGCGGGCCTCATCTCGAGTTGATTTGGGGCCCATGGAGCTCCTTCGCGATGCTGGTGTGACCT
CAGGGTCCCTCTCTACTTGTGACAGTGTTTTTGCGGACTGTCTCGACATCCATCAAGCAAGTCGAGGCTCCTGACATGTTTCAGGGGGCTCACGGAATTGCTCTGCA
TGCAGTGCAGGGGAATCGGGCCTCATCTCGCTTGGAGGGGAAGTCTC

iii. SRR26335613__GENOMAD__Virus.1 (VSLC-1) intergenic repeated components

>IGRC$_1$
ACTGTATTCAAGCCACTGCAAGGAAAC
>IGRC$_2$
CCGGCCTCCTTTCGAGTCAGGGCTTCTCGGTGTATATGCCCATTGAGGCAGCAATCTCAGGGTCCCTCCCTCATACCTATTGCT
GAGAGAAGCCTCCTCTTGAGGGGCTTGTGGAAAGGTGGCCTACGTCTTGAGTCTAAGCCAGGGAATCAGCTCTCATCTCGAGG
CAATTTGGGGTACACGGAGCAGTCCTCGAGTTGCTCTGCTGAACTTGGTACTCCTCTAGACTTGGCCCGTTGTTCTCGGGGAA
CCTCTGGAGTTGCCTAAAGGGAGTCAAGCCTCAGGTCGTGTTTGATGGGGAACGGGGGATGGCTCTGGAGTCAATGCAGGGG
AAGCGGGCCTCATCTCGAGTTGATTTGGGGCCCATGGAGCTCCTTCGCGATGCTGGTGTGACCTCAGGGTCCCTCTCTACTTG
TGACAGTGTTTTT-GCGGACTGTCTCGACATCCATCAAGCAAGTCGAGGCTCCTGAC
>IGRC$_3$
ATGTTTCAGGGGGCTCACGGAATTGCTCTG
>IGRC$_4$
CATGCAGTGCAGGGGAATCGGGCCTC--ATCTCGCTTGG

iv. SRR26335613__GENOMAD__Virus.1 (VSLC-1) intergenic repeated components (MSA)

>g12-g13
ACTGTATTCAAGCCACTGCAAGGAAACCCGGCCTCCTTTCGAGTCAGGGCTTCTCGGTGTATATGCCCATTGAGGCAGCAATCT
CAGGGTCCCTCCCTCATACCTATTGCTGAGAGAAGCCTCCTCTTGAGGGGCTTGTGGAAAGGTGGCCTACGTCTTGAGTCTAA
GCCAGGGAATCAGCTCTCATCTCGAGGCAATTTGGGGTACACGGAGCAGTCCTCGAGTTGCTCTGCTGAACTTGGTACTCCTC
TAGACTTGGCCCGTTGTTCTCGGGGAACCTCTGGAGTTGCCTAAAGGGAGTCAAGCCTCAGGTCGTGTTTGATGGGGAACGGG
GGATGGCTCTGGAGTCAATGCAGGGGAAGCGGGCCTCATCTCGAGTTGATTTGGGGCCCATGGAGCTCCTTCGCGATGCTGG
TGTGACCTCAGGGTCCCTCTCTACTTGTGACAGTGTTTTT-GCGGACTGTCTCGACATCCATCAAGCAAGTCGAGGCTCCTGAC-
----

>g15-g16
ACTGTATTCAAGCCACTGCAAGGAAACCCGGCCTCCTTTCGAGTCAGGGCTTCTCGGTGTATATGCCCATTGAGGCAGCAATCT
CAGGGTCCCTCCCTCATACCTATTGCTGAGAGAAGCCTCCTCTTGAGGGGCTTGTGGAAAGGTGGCCTACGTCTTGAGTCTAA
GCCAGGGAATCAGCTCTCATCTCGAGGCAATTTGGGGTACACGGAGCAGTCCTCGAGTTGCTCTGCTGAACTTGGTACTCCTC
TAGACTTGGCCCGTTGTTCTCGGGGAACCTCTGGAGTTGCCTAAAGGGAGTCAAGCCTCAGGTCGTGTTTGATGGGGAACGGG
GGATGGCTCTGGAGTCAATGCAGGGGAAGCGGGCCTCATCTCGAGTTGATTTGGGGCCCATGGAGCTCCTTCGCGATGCTGG
TGTGACCTCAGGGTCCCTCTCTACTTGTGACAGTGTTTTT-GCGGACTGTCTCGACATCCATCAAGCAAGTCGAGGCTCCTGAC-
----

>g26-g27
ACTGTATTCAAGCCACTGCAAGGAAACCCGGCCTCCTTTCGAGTCAGGGCTTCTCGGTGTATATGCCCATTGAGGCAGCAATCT
CAGGGTCCCTCCCTCATACCTATTGCTGAGAGAAGCCTCCTCTTGAGGGGCTTGTGGAAAGGTGGCCTACGTCTTGAGTCTAA
GCCAGGGAATCAGCTCTCATCTCGAGGCAATTTGGGGTACACGGAGCAGTCCTCGAGTTGCTCTGCTGAACTTGGTACTCCTC
TAGACTTGGCCCGTTGTTCTCGGGGAACCTCTGGAGTTGCCTAAAGGGAGTCAAGCCTCAGGTCGTGTTTGATGGGGAACGGG
GGATGGCTCTGGAGTCAATGCAGGGGAAGCGGGCCTCATCTCGAGTTGATTTGGGGCCCATGGAGCTCCTTCGCGATGCTGG
TGTGACCTCAGGGTCCCTCTCTACTTGTGACAGTGTTTTT-GCGGACTGTCTCGACATCCATCAAGCAAGTCGAGGCTCCTGAC-
----

>g16-g17
ACTGTATTCAAGCCACTGCAAGGAAACCCGGCCTCCTTTCGAGTCAGGGCTTCTCGGTGTATATGCCCATTGAGGCAGCAATCT
CAGGGTCCCTCCCTCATACCTATTGCTGAGAGAAGCCTCCTCTTGAGGGGCTTGTGGAAAGGTGGCCTACGTCTTGAGTCTAA
GCCAGGGAATCAGCTCTCATCTCGAGGCAATTTGGGGTACACGGAGCAGTCCTCGAGTTGCTCTGCTGAACTTGGTACTCCTC
TAGACTTGGCCCGTTGTTCTCGGGGAACCTCTGGAGTTGCCTAAAGGGAGTCAAGCCTCAGGTCGTGTTTGATGGGGAACGGG
GGATGGCTCTGGAGTCAATGCAGGGGAAGCGGGCCTCATCTCGAGTTGATTTGGGGCCCATGGAGCTCCTTCGCGATGCTGG
TGTGACCTCAGGGTCCCTCTCTACTTGTGACAGTGTTTTT-GCGGACTGTCTCGACATCCATCAAGCAAGTCGAGGCTCCTGAC-
----

>g23-g24
ACTGTATTCAAGCCACTGCAAGGAAACCCGGCCTCCTTTCGAGTCAGGGCTTCTCGGTGTATATGCCCATTGAGGCAGCAATCT
CAGGGTCCCTCCCTCATACCTATTGCTGAGAGAAGCCTCCTCTTGAGGGGCTTGTGGAAAGGTGGCCTACGTCTTGAGTCTAA
GCCAGGGAATCAGCTCTCATCTCGAGGCAATTTGGGGTACACGGAGCAGTCCTCGAGTTGCTCTGCTGAACTTGGTACTCCTC
TAGACTTGGCCCGTTGTTCTCGGGGAACCTCTGGAGTTGCCTAAAGGGAGTCAAGCCTCAGGTCGTGTTTGATGGGGAACGGG
GGATGGCTCTGGAGTCAATGCAGGGGAAGCGGGCCTCATCTCGAGTTGATTTGGGGCCCATGGAGCTCCTTCGCGATGCTGG
TGTGACCTCAGGGTCCCTCTCTACTTGTGACAGTGTTTTT-GCGGACTGTCTCGACATCCATCAAGCAAGTCGAGGCTCCTGAC-
----

>g6-g7
ACTGTATTCAAGCCACTGCAAGGAAACCCGGCCTCCTTTCGAGTCAGGGCTTCTCGGTGTATATGCCCATTGAGGCAGCAATCT
CAGGGTCCCTCCCTCATACCTATTGCTGAGAGAAGCCTCCTCTTGAGGGGCTTGTGGAAAGGTGGCCTACGTCTTGAGTCTAA
GCCAGGGAATCAGCTCTCATCTCGAGGCAATTTGGGGTACACGGAGCAGTCCTCGAGTTGCTCTGCTGAACTTGGTACTCCTC
TAGACTTGGCCCGTTGTTCTCGGGGAACCTCTGGAGTTGCCTAAAGGGAGTCAAGCCTCAGGTCGTGTTTGATGGGGAACGGG
GGATGGCTCTGGAGTCAATGCAGGGGAAGCGGGCCTCATCTCGAGTTGATTTGGGGCCCATGGAGCTCCTTCGCGATGCTGG
TGTGACCTCAGGGTCCCTCTCTACTTGTGACAGTGTTTTT-GCGGACTGTCTCGACATCCATCAAGCAAGTCGAGGCTCCTGAC-
----

>g1-g2
ACTGTATTCAAGCCACTGCAAGGAAACCCGGCCTCCTTTCGAGTCAGGGCTTCTCGGTGTATATGCCCATTGAGGCAGCAATCT
CAGGGTCCCTCCCTCATACCTATTGCTGAGAGAAGCCTCCTCTTGAGGGGCTTGTGGAAAGGTGGCCTACGTCTTGAGTCTAA
GCCAGGGAATCAGCTCTCATCTCGAGGCAATTTGGGGTACACGGAGCAGTCCTCGAGTTGCTCTGCTGAACTTGGTACTCCTC
TAGACTTGGCCCGTTGTTCTCGGGGAACCTCTGGAGTTGCCTAAAGGGAGTCAAGCCTCAGGTCGTGTTTGATGGGGAACGGG
GGATGGCTCTGGAGTCAATGCAGGGGAAGCGGGCCTCATCTCGAGTTGATTTGGGGCCCATGGAGCTCCTTCGCGATGCTGG
TGTGACCTCAGGGTCCCTCTCTACTTGTGACAGTGTTTTT-GCGGACTGTCTCGACATCCATCAAGCAAGTCGAGGCTCCTGAC-
----

>g14-g15
ACTGTATTCAAGCCACTGCAAGGAAACCCGGCCTCCTTTCGAGTCAGGGCTTCTCGGTGTATATGCCCATTGAGGCAGCAATCT
CAGGGTCCCTCCCTCATACCTATTGCTGAGAGAAGCCTCCTCTTGAGGGGCTTGTGGAAAGGTGGCCTACGTCTTGAGTCTAA
GCCAGGGAATCAGCTCTCATCTCGAGGCAATTTGGGGTACACGGAGCAGTCCTCGAGTTGCTCTGCTGAACTTGGTACTCCTC
TAGACTTGGCCCGTTGTTCTCGGGGAACCTCTGGAGTTGCCTAAAGGGAGTCAAGCCTCAGGTCGTGTTTGATGGGGAACGGG
GGATGGCTCTGGAGTCAATGCAGGGGAAGCGGGCCTCATCTCGAGTTGATTTGGGGCCCATGGAGCTCCTTCGCGATGCTGG
TGTGACCTCAGGGTCCCTCTCTACTTGTGACAGTGTTTTT-GCGGACTGTCTCGACATCCATCAAGCAAGTCGAGGCTCCTGAC-
----

>g9-g10
ACTGTATTCAAGCCACTGCAAGGAAACCCGGCCTCCTTTCGAGTCAGGGCTTCTCGGTGTATATGCCCATTGAGGCAGCAATCT
CAGGGTCCCTCCCTCATACCTATTGCTGAGAGAAGCCTCCTCTTGAGGGGCTTGTGGAAAGGTGGCCTACGTCTTGAGTCTAA
GCCAGGGAATCAGCTCTCATCTCGAGGCAATTTGGGGTACACGGAGCAGTCCTCGAGTTGCTCTGCTGAACTTGGTACTCCTC
TAGACTTGGCCCGTTGTTCTCGGGGAACCTCTGGAGTTGCCTAAAGGGAGTCAAGCCTCAGGTCGTGTTTGATGGGGAACGGG
GGATGGCTCTGGAGTCAATGCAGGGGAAGCGGGCCTCATCTCGAGTTGATTTGGGGCCCATGGAGCTCCTTCGCGATGCTGG
TGTGACCTCAGGGTCCCTCTCTACTTGTGACAGTGTTTTT-GCGGACTGTCTCGACATCCATCAAGCAAGTCGAGGCTCCTGAC-
----

>g11-g12
ACTGTATTCAAGCCACTGCAAGGAAACCCGGCCTCCTTTCGAGTCAGGGCTTCTCGGTGTATATGCCCATTGAGGCAGCAATCT
CAGGGTCCCTCCCTCATACCTATTGCTGAGAGAAGCCTCCTCTTGAGGGGCTTGTGGAAAGGTGGCCTACGTCTTGAGTCTAA
GCCAGGGAATCAGCTCTCATCTCGAGGCAATTTGGGGTACACGGAGCAGTCCTCGAGTTGCTCTGCTGAACTTGGTACTCCTC
TAGACTTGGCCCGTTGTTCTCGGGGAACCTCTGGAGTTGCCTAAAGGGAGTCAAGCCTCAGGTCGTGTTTGATGGGGAACGGG
GGATGGCTCTGGAGTCAATGCAGGGGAAGCGGGCCTCATCTCGAGTTGATTTGGGGCCCATGGAGCTCCTTCGCGATGCTGG
TGTGACCTCAGGGTCCCTCTCTACTTGTGACAGTGTTTTT-GCGGACTGTCTCGACATCCATCAAGCAAGTCGAGGCTCCTGAC-
----

>g18-g19
ACTGTATTCAAGCCACTGCAAGGAAACCCGGCCTCCTTTCGAGTCAGGGCTTCTCGGTGTATATGCCCATTGAGGCAGCAATCT
CAGGGTCCCTCCCTCATACCTATTGCTGAGAGAAGCCTCCTCTTGAGGGGCTTGTGGAAAGGTGGCCTACGTCTTGAGTCTAA
GCCAGGGAATCAGCTCTCATCTCGAGGCAATTTGGGGTACACGGAGCAGTCCTCGAGTTGCTCTGCTGAACTTGGTACTCCTC
TAGACTTGGCCCGTTGTTCTCGGGGAACCTCTGGAGTTGCCTAAAGGGAGTCAAGCCTCAGGTCGTGTTTGATGGGGAACGGG
GGATGGCTCTGGAGTCAATGCAGGGGAAGCGGGCCTCATCTCGAGTTGATTTGGGGCCCATGGAGCTCCTTCGCGATGCTGG
TGTGACCTCAGGGTCCCTCTCTACTTGTGACAGTGTTTTT-GCGGACTGTCTCGACATCCATCAAGCAAGTCGAGGCTCCTGAC-
----

>g3-g4
ACTGTATTCAAGCCACTGCAAGGAAACCCGGCCTCCTTTCGAGTCAGGGCTTCTCGGTGTATATGCCCATTGAGGCAGCAATCT
CAGGGTCCCTCCCTCATACCTATTGCTGAGAGAAGCCTCCTCTTGAGGGGCTTGTGGAAAGGTGGCCTACGTCTTGAGTCTAA
GCCAGGGAATCAGCTCTCATCTCGAGGCAATTTGGGGTACACGGAGCAGTCCTCGAGTTGCTCTGCTGAACTTGGTACTCCTC
TAGACTTGGCCCGTTGTTCTCGGGGAACCTCTGGAGTTGCCTAAAGGGAGTCAAGCCTCAGGTCGTGTTTGATGGGGAACGGG
GGATGGCTCTGGAGTCAATGCAGGGGAAGCGGGCCTCATCTCGAGTTGATTTGGGGCCCATGGAGCTCCTTCGCGATGCTGG
TGTGACCTCAGGGTCCCTCTCTACTTGTGACAGTGTTTTT-GCGGACTGTCTCGACATCCATCAAGCAAGTCGAGGCTCCTGAC-
----

>g13-g14
ACTGTATTCAAGCCACTGCAAGGAAACCCGGCCTCCTTTCGAGTCAGGGCTTCTCGGTGTATATGCCCATTGAGGCAGCAATCT
CAGGGTCCCTCCCTCATACCTATTGCTGAGAGAAGCCTCCTCTTGAGGGGCTTGTGGAAAGGTGGCCTACGTCTTGAGTCTAA
GCCAGGGAATCAGCTCTCATCTCGAGGCAATTTGGGGTACACGGAGCAGTCCTCGAGTTGCTCTGCTGAACTTGGTACTCCTC
TAGACTTGGCCCGTTGTTCTCGGGGAACCTCTGGAGTTGCCTAAAGGGAGTCAAGCCTCAGGTCGTGTTTGATGGGGAACGGG
GGATGGCTCTGGAGTCAATGCAGGGGAAGCGGGCCTCATCTCGAGTTGATTTGGGGCCCATGGAGCTCCTTCGCGATGCTGG
TGTGACCTCAGGGTCCCTCTCTACTTGTGACAGTGTTTTT-GCGGACTGTCTCGACATCCATCAAGCAAGTCGAGGCTCCTGAC-
----

>g19-g20
ACTGTATTCAAGCCACTGCAAGGAAACCCGGCCTCCTTTCGAGTCAGGGCTTCTCGGTGTATATGCCCATTGAGGCAGCAATCT
CAGGGTCCCTCCCTCATACCTATTGCTGAGAGAAGCCTCCTCTTGAGGGGCTTGTGGAAAGGTGGCCTACGTCTTGAGTCTAA
GCCAGGGAATCAGCTCTCATCTCGAGGCAATTTGGGGTACACGGAGCAGTCCTCGAGTTGCTCTGCTGAACTTGGTACTCCTC
TAGACTTGGCCCGTTGTTCTCGGGGAACCTCTGGAGTTGCCTAAAGGGAGTCAAGCCTCAGGTCGTGTTTGATGGGGAACGGG
GGATGGCTCTGGAGTCAATGCAGGGGAAGCGGGCCTCATCTCGAGTTGATTTGGGGCCCATGGAGCTCCTTCGCGATGCTGG
TGTGACCTCAGGGTCCCTCTCTACTTGTGACAGTGTTTTT-GCGGACTGTCTCGACATCCATCAAGCAAGTCGAGGCTCCTGAC-
----

>g20-g21
ACTGTATTCAAGCCACTGCAAGGAAACCCGGCCTCCTTTCGAGTCAGGGCTTCTCGGTGTATATGCCCATTGAGGCAGCAATCT
CAGGGTCCCTCCCTCATACCTATTGCTGAGAGAAGCCTCCTCTTGAGGGGCTTGTGGAAAGGTGGCCTACGTCTTGAGTCTAA
GCCAGGGAATCAGCTCTCATCTCGAGGCAATTTGGGGTACACGGAGCAGTCCTCGAGTTGCTCTGCTGAACTTGGTACTCCTC
TAGACTTGGCCCGTTGTTCTCGGGGAACCTCTGGAGTTGCCTAAAGGGAGTCAAGCCTCAGGTCGTGTTTGATGGGGAACGGG
GGATGGCTCTGGAGTCAATGCAGGGGAAGCGGGCCTCATCTCGAGTTGATTTGGGGCCCATGGAGCTCCTTCGCGATGCTGG
TGTGACCTCAGGGTCCCTCTCTACTTGTGACAGTGTTTTT-GCGGACTGTCTCGACATCCATCAAGCAAGTCGAGGCTCCTGAC-
----

>U53517.1

CCGGCCTCCTTTCGAGTCAGGGCTTCTCGGTGTATATGCCCATTGAGGCAGCAATCTCAGGGTCCCTCCTTCATACCTATTGCT
GAGAGAAGCCTCCTCTTGAGGGGCTTGTGGAAAGGTGGCCTACGTATTGAGTCTAAGCCAGGGAATCAGCTCTCATCTCGAGG
CAATTTGGGGTACACGGAGCAGTCCTCGAGTTGCTCTGCTGAACTTGGTACTCGTCTAGACTTGGCCCGTTGTTCTCCAGGAAC
CTCTGGAGTTGCCTAAAGGGAGTCAAGCCTCAGGTCGTGTTTGATGGGGAACTGGGGATGGCTCTGGAGTCAATGCAGGGGA
AGCGGGCCTCATCTCAAGTTGATTTGGGGCCCATGGAGCTCCTTCGCGATGCTGGTGTGACCTCAGGGTCCCTCTCTACTTGT
GACAGTGTTTTTGGCGGACTGTCTCGACATCCATCAAGCAAGTCGAGGCTCCTGACATGTTTCAGGGGTCTCACGGAATTGNTA

TGCATGCAGTGCAGGGGAATCGGGCCTCATATCTCGCTTGGAGGGGGAAGTCTCATGGTTTTTCTCGAGTTCCGGCCCGGACC
TGGGGTACATTCTCGACTTTACGGCGGGGATGCCCCTTCAACCCTCGTGTTTGNTCAACGAAGTCAGGAGTCCTGTTGAGTTAC
GAGGGACACCTCAGGAGTCTCTTCGAGGCTTGGCAGGGCAAAAGGGACGCTTCTCGAGGTGAGTCGGGAGACCCAGGGTCC
CTTTCCAGTAGCCACAGGGATACTGGGATTCCTGTCAATGTTCAAGAGGAGTCAGGCTCCGTCAAATTTTGAAANAGTGAGCTC
TGCGTGCTTCTCGAGGTGTCAGAGGCATGTGAGGCATTCCGTCGAGAGGAGTCGGGGACCTAGGGCTTTCTCTAGGGACTCC
ACAGGTGGTGCAGACATCCCTTCACCTTGTGAGATGAAAGACTAGACTGTATTCAAGCCACTGCAAGGAAACCCGG
>g8-g9
ACTGTATTCAAGCCACTGCAAGGAAACCCGGCCTCCTTTCGAGTCAGGGCTTCTCGGTGTATATGCCCATTGAGGCAGCAATCT
CAGGGTCCCTCCCTCATACCTATTGCTGAGAGAAGCCTCCTCTTGAGGGGCTTGTGGAAAGGTGGCCTACGTCTTGAGTCTAA
GCCAGGGAATCAGCTCTCATCTCGAGGCAATTTGGGGTACACGGAGCAGTCCTCGAGTTGCTCTGCTGAACTTGGTACTCGTC
TAGACTTGGCCCGTTGTTCTCCGGGAACCTCTGGAGTTGCCTAAAGGGAGTCAAGCCTCAGGTCGTGTTTGATGGGGAACTGG
GGATGGCTCTGGAGTCAATGCAGGGGAAGCGGGCCTCATCTCGAGTTGATTTGGGGCCCATGGAGCTCCTTCGCGATGCTGG
TGTGACCTCAGGGTCCCTCTCTACTTGTGACAGTGTTTTT-GCGGACTGTCTCGACATCCATCAAGCAAGTCGAGGCTCCTGAC-
----

>g10-g11
ACTGTATTCAAGCCACTGCAAGGAAACCCGGCCTCCTTTCGAGTCAGGGCTTCTCGGTGTATATGCCCATTGAGGCAGCAATCT
CAGGGTCCCTCCCTCATACCTATTGCTGAGAGAAGCCTCCTCTTGAGGGGCTTGTGGAAAGGTGGCCTACGTCTTGAGTCTAA
GCCAGGGAATCAGCTCTCATCTCGAGGCAATTTGGGGTACACGGAGCAGTCCTCGAGTTGCTCTGCTGAACTTGGTACTCCTC
TAGACTTGGCCCGTTGTTCTCCGGGAACCTCTGGAGTTGCCTAAAGGGAGTCAAGCCTCAGGTCGTGTTTGATGGGGAACAGG
GGATGGCTCTGGAGTCAATGCAGGGGAAGCGGGCCTCATCTCGAGTTGATTTGGGGCCCATGGAGCTCCTTCGCGATGCTGG
TGTGACCTCAGGGTCCCTCTCTACTTGTGACAGTGTTTTT-GCGGACTGTCTCGACATCCATCAAGCAAGTCGAGGCTCCTGAC-
----

>g5-g6
ACTGTATTCAAGCCACTGCAAGGAAACCCGGCCTCCTTTCGAGTCAGGGCTTCTCGGTGTATATGCCCATTGAGGCAGCAATCT
CAGGGTCCCTCCCTCATACCTATTGCTGAGAGAAGCCTCCTCTTGAGGGGCTTGTGGAAAGGTGGCCTACGTCTTGAGTCTAA
GCCAGGGAATCAGCTCTCATCTCGAGGCAATTTGGGGTACACGGAGCAGTCCTCGAGTTGCTCTGCTGAACTTGGTACTCCTC
TAGACTTGGCCCGTTGTTCTCCGGGAACCTCTGGAGTTGCCTAAAGGGAGTCAAGCCTCAGGTCGTGTTTGATGGGGAACGGG
GGATGGCTCTGGAGTCAATGCAGGGGAAGCGGGCCTCATCTCGAGTTGATTTGGGGCCCATGGAGCTCCTTCGCGATGCTGG
TGTGACCTCAGGGTCCCTCTCTACTTGTGACAGTGTTTTT-GCGGACTGTCTCGACATCCATCAAGCAAGTCGAGGCTCCTGAC-
----

>g4-g5
ACTGTATTCAAGCCACTGCAAGGAAACCCGGCCTCCTTTCGAGTCAGGGCTTCTCGGTGTATATGCCCATTGAGGCAGCAATCT
CAGGGTCCCTCCCTCATACCTATTGCTGAGAGAAGCCTCCTCTTGAGGGGCTTGTGGAAAGGTGGCCTACGTCTTGAGTCTAA
GCCAGGGAATCAGCTCTCATCTCGAGGCAATTTGGGGTACACGGAGCAGTCCTCGAGTTGCTCTGCTGAACTTGGTACTCCTC
TAGACTTGGCCCGTTGTTCTCCGGGAACCTCTGGAGTTGCCTAAAGGGAGTCAAGCCTCAGGTCGTGTTTGATGGGGAACGGG
GGATGGCTCTGGAGTCAATGCAGGGGAAGCGGGCCTCATCTCGAGTTGATTTGGGGCCCATGGAGCTCCTTCGCGATGCTGG
TGTGACCTCAGGGTCCCTCTCTACTTGTGACAGTGTTTTT-GCGGACTGTCTCGACATCCATCAAGCAAGTCGAGGCTCCTGAC-
----

>g25-g26
ACTGTATTCAAGCCACTGCAAGGAAACCCGGCCTCCTTTCGAGTCAGGGCTTCTCGGTGTATATGCCCATTGAGGCAGCAATCT
CAGGGTCCCTCCCTCATACCTATTGCTGAGAGAAGCCTCCTCTTGAGGGGCTTGTGGAAAGGTGGCCTACGTCTTGAGTCTAA
GCCAGGGAATCAGCTCTCATCTCGAGGCAATTTGGGGTACACGGAGCAGTCCTCGAGTTGCTCTGCTGAACTTGGTACTCCTC
TAGACTTGGCCCGTTGTTCTCCGGGAACCTCTGGAGTTGCCTAAAGGGAGTCAAGCCTCAGGTCGTGTTTGATGGGGAACGGG
GGATGGCTCTGGAGTCAATGCAGGGGAAGCGGGCCTCATCTCGAGTTGATTTGGGGCCCATGGAGCTCCTTCGCGATGCTGG
TGTGACCTCAGGGTCCCTCTCTACTTGTGACAGTGTTTTT-
GCGGACTGTCTCGACATCCATCAAGCAAGTCGAGGCTCCTGACATGTTTCAGGGGGCTCACGGAATTGCTCTG-----------------------
----

--C
>g17-g18
ACTGTATTCAAGCCACTGCAAGGAAACCCGGCCTCCTTTCGAGTCAGGGCTTCTCGGTGTATATGCCCATTGAGGCAGCAATCT
CAGGGTCCCTCCCTCATACCTATTGCTGAGAGAAGCCTCCTCTTGAGGGGCTTGTGGAAAGGTGGCCTACGTCTTGAGTCTAA
GCCAGGGAATCAGCTCTCATCTCGAGGCAATTTGGGGTACACGGAGCAGTCCTCGAGTTGCTCTGCTGAACTTGGTACTCCTC
TAGACTTGGCCCGTTGTTCTCG-
GGAACCTCTGGAGTTGCCTAAAGGGAGTCAAGCCTCAGGTCGTGTTTGATGGGGAACGGGGGATGGCTCTGGAGTCAATGCA
GGGGAAGCGGGCCTCATCTCGAGTTGATTTGGGGCCCATGGAGCTCCTTCGCGATGCTGGTGTGACCTCAGGGTCCCTCTCT
ACTTGTGACAGTGTTTTT-GCGGACTGTCTCGACATCCATCAAGCAAGTCGAGGCTCCTGAC--
----

>g7-g8

ACTGTATTCAAGCCACTGCAAGGAAACCCGGCCTCCTTTCGAGTCAGGGCTTCTCGGTGTATATGCCCATTGAGGCAGCAATCT
CAGGGTCCCTCCCTCATACCTATTGCTGAGAGAAGCCTCCTCTTGAGGGGCTTGTGGAAAGGTGGCCTACGTCTTGAGTCTAA
GCCAGGGAATCAGCTCTCATCTCGAGGCAATTTGGGGTACACGGAGCAGTCCTCGAGTTGCTCTGCTGAACTTGGTACTCCTC
TAGACTTGGCCCGTTGTTCTCGGGGAACCTCTGGAGTTGCCTAAAGGGAGTCAAGCCTCAGGTCGTGTTTGATGGGGAACGGG
GGATGGCTCTGGAGTCAATGCAGGGGAAGCGGGCCTCATCTCGAGTTGATTTGGGGCCCATGGAGCTCCTTCGCGATGCTGG
TGTGACCTCAGGGTCCCTCTCTACTTGTGACAGTGTTTTT-
GCGGACTGTCTCGACATCCATCAAGCAAGTCGAGGCTCCTGACATGTTTCAGGGGGCTCACGGAATTGCTCTG---------------------------
----

---C
>g24-g25
ACTGTATTCAAGCCACTGCAAGGAAACCCGGCCTCCTTTCGAGTCAGGGCTTCTCGGTGTATATGCCCATTGAGGCAGCAATCT
CAGGGTCCCTCCCTCATACCTATTGCTGAGAGAAGCCTCCTCTTGAGGGGCTTGTGGAAAGGTGGCCTACGTCTTGAGTCTAA
GCCAGGGAATCAGCTCTCATCTCGAGGCAATTTGGGGTACACGGAGCAGTCCTCGAGTTGCTCTGCTGAACTTGGTACTCCTC
TAGACTTGGCCCGTTGTTCTCGGGGAACCTCTGGAGTTGCCTAAAGGGAGTCAAGCCTCAGGTCGTGTTTGATGGGGAACGGG
GGATGGCTCTGGAGTCAATGCAGGGGAAGCGGGCCTCATCTCGAGTTGATTTGGGGCCCATGGAGCTCCTTCGCGATGCTGG
TGTGACCTCAGGGTCCCTCTCTACTTGTGACAGTGTTTTT-
GCGGACTGTCTCGACATCCATCAAGCAAGTCGAGGCTCCTGACATGTTTCAGGGGGCTCACGGAATTGCTCTG---------------------------
----

---C
>g2-g3
ACTGTATTCAAGCCACTGCAAGGAAACCCGGCCTCCTTTCGAGTCAGGGCTTCTCGGTGTATATGCCCATTGAGGCAGCAATCT
CAGGGTCCCTCCCTCATACCTATTGCTGAGAGAAGCCTCCTCTTGAGGGGCTTGTGGAAAGGTGGCCTACGTCTTGAGTCTAA
GCCAGGGAATCAGCTCTCATCTCGAGGCAATTTGGGGTACACGGAGCAGTCCTCGAGTTGCTCTGCTGAACTTGGTACTCCTC
TAGACTTGGCCCGTTGTTCTCGGGGAACCTCTGGAGTTGCCTAAAGGGAGTCAAGCCTCAGGTCGTGTTTGATGGGGAACGGG
GGATGGCTCTGGAGTCAATGCAGGGGAAGCGGGCCTCATCTCGAGTTGATTTGGGGCCCATGGAGCTCCTTCGCGATGCTGG
TGTGACCTCAGGGTCCCTCTCTACTTGTGACAGTGTTTTT-
GCGGACTGTCTCGACATCCATCAAGCAAGTCGAGGCTCCTGACATGTTTCAGGGGGCTCACGGAATTGCTCTG---------------------------
----

---C
>g21-g22
ACTGTATTCAAGCCACTGCAAGGAAACCCGGCCTCCTTTCGAGTCAGGGCTTCTCGGTGTATATGCCCATTGAGGCAGCAATCT
CAGGGTCCCTCCCTCATACCTATTGCTGAGAGAAGCCTCCTCTTGAGGGGCTTGTGGAAAGGTGGCCTACGTCTTGAGTCTAA
GCCAGGGAATCAGCTCTCATCTCGAGGCAATTTGGGGTACACGGAGCAGTCCTCGAGTTGCTCTGCTGAACTTGGTACTCCTC
TAGACTTGGCCCGTTGTTCTCGGGGAACCTCTGGAGTTGCCTAAAGGGAGTCAAGCCTCAGGTCGTGTTTGATGGGGAACGGG
GGATGGCTCTGGAGTCAATGCAGGGGAAGCGGGCCTCATCTCGAGTTGATTTGGGGCCCATGGAGCTCCTTCGCGATGCTGG
TGTGACCTCAGGGTCCCTCTCTACTTGTGACAGTGTTTTT-
GCGGACTGTCTCGACATCCATCAAGCAAGTCGAGGCTCCTGACATGTTTCAGGGGGCTCACGGAATTGCTCTG---------------------------
----

---C
>g22-g23
ACTGTATTCAAGCCACTGCAAGGAAACCCGGCCTCCTTTCGAGTCAGGGCTTCTCGGTGTATATGCCCATTGAGGCAGCAATCT
CAGGGTCCCTCCCTCATACCTATTGCTGAGAGAAGCCTCCTCTTGAGGGGCTTGTGGAAAGGTGGCCTACGTCTTGAGTCTAA
GCCAGGGAATCAGCTCTCATCTCGAGGCAATTTGGGGTACACGGAGCAGTCCTCGAGTTGCTCTGCTGAACTTGGTACTCCTC
TAGACTTGGCCCGTTGTTCTCGGGGAACCTCTGGAGTTGCCTAAAGGGAGTCAAGCCTCAGGTCGTGTTTGATGGGGAACGGG
GGATGGCTCTGGAGTCAATGCAGGGGAAGCGGGCCTCATCTCGAGTTGATTTGGGGCCCATGGAGCTCCTTCGCGATGCTGG
TGTGACCTCAGGGTCCCTCTCTACTTGTGACAGTGTTTTT-
GCGGACTGTCTCGACATCCATCAAGCAAGTCGAGGCTCCTGACATGTTTCAGGGGGCTCACGGAATTGCTCTG---------------------------
----

---C
>g27-g28
ACTGTATTCAAGCCACTGCAAGGAAACCCGGCCTCCTTTCGAGTCAGGGCTTCTCGGTGTATATGCCCATTGAGGCAGCAATCT
CAGGGTCCCTCCCTCATACCTATTGCTGAGAGAAGCCTCCTCTTGAGGGGCTTGTGGAAAGGTGGCCTACGTCTTGAGTCTAA
GCCAGGGAATCAGCTCTCATCTCGAGGCAATTTGGGGTACACGGAGCAGTCCTCGAGTTGCTCTGCTGAACTTGGTACTCCTC
TAGACTTGGCCCGTTGTTCTCGGGGAACCTCTGGAGTTGCCTAAAGGGAGTCAAGCCTCAGGTCGTGTTTGATGGGGAACGGG
GGATGGCTCTGGAGTCAATGCAGGGGAAGCGGGCCTCATCTCGAGTTGATTTGGGGCCCATGGAGCTCCTTCGCGATGCTGG
TGTGACCTCAGGGTCCCTCTCTACTTGTGACAGTGTTTTT-
GCGGACTGTCTCGACATCCATCAAGCAAGTCGAGGCTCCTGACATGTTTCAGGGGGCTCACGGAATTGCTCTGCATGCAGTGC
AGGGGAATCGGGCCTC--ATCTCGCTTGG--

---AGGGGAAGTCTC

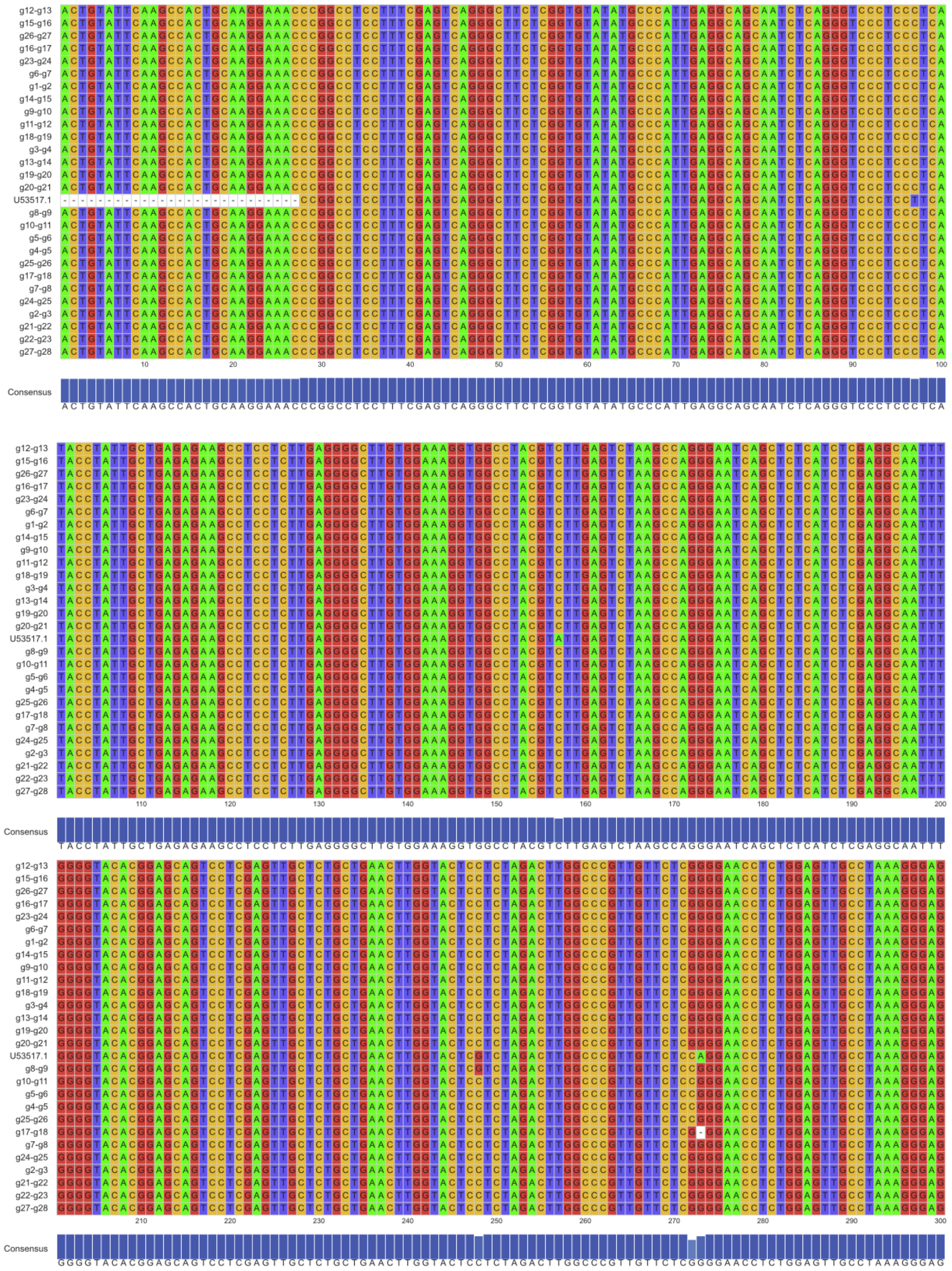
g12-g13
g15-g16
g26-g27
g16-g17
g23-g24
g6-g7
g1-g2
g14-g15
g9-g10
g11-g12
g18-g19
g3-g4
g13-g14
g19-g20
g20-g21
U53517.1
g8-g9
g10-g11
g5-g6
g4-g5
g25-g26
g17-g18
g7-g8
g24-g25
g2-g3
g21-g22
g22-g23
g27-g28
10 20 30 40 50 60 70 80 90 100
Consensus
ACTGTATTCAAGCCACTGCAAGGAAACCCGGCCTCCTTTCGAGTCAGGGCTTCTCGGTGTATATGCCCATTGAGGCAGCAATCTCAGGGTCCCTCCCTCA
110 120 130 140 150 160 170 180 190 200
Consensus
TACCTATTGCTGAGAGAAGCCTCCTCTTGAGGGGCTTGTGGAAAGGTGGCCTACGTCTTGAGTCTAAGCCAGGGAATCAGCTCTCATCTCGAGGCAATTT
210 220 230 240 250 260 270 280 290 300
Consensus
GGGGTACACGGAGCAGTCCTCGAGTTGCTCTGCTGAACTTGGTACTCCTCTAGACTTGGCCCGTTGTTCTCGGGGAACCTCTGGAGTTGCCTAAAGGGAG

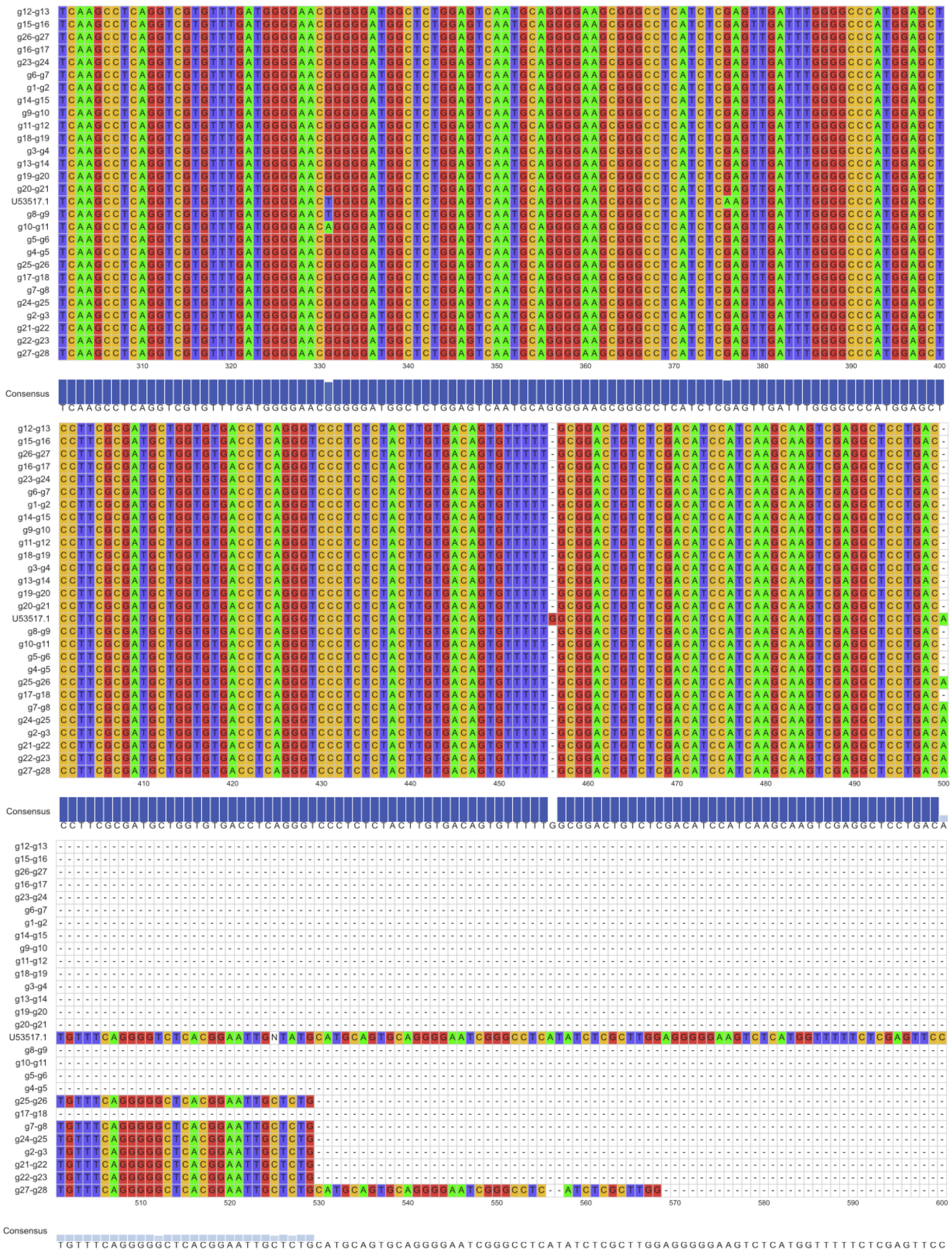
g12-g13
g15-g16
g26-g27
g16-g17
g23-g24
g6-g7
g1-g2
g14-g15
g9-g10
g11-g12
g18-g19
g3-g4
g13-g14
g19-g20
g20-g21
U53517.1
g8-g9
g10-g11
g5-g6
g4-g5
g25-g26
g17-g18
g7-g8
g24-g25
g2-g3
g21-g22
g22-g23
g27-g28
Consensus
TCAAGCCTCAGGTCGTGTTTGATGGGGAACGGGGGATGGCTCTGGAGTCAATGCAGGGGAAGCGGGCCTCATCTCGAGTTGATTTGGGGCCCATGGAGCT
Consensus
CCTTCGCGATGCTGGTGTGACCTCAGGGTCCCTCTCTACTTGTGACAGTGTTTTTGGCGGACTGTCTCGACATCCATCAAGCAAGTCGAGGCTCCTGACA
Consensus
TGTTTCAGGGGGCTCACGGAATTGCTCTGCATGCAGTGCAGGGGAATCGGGCCTCATATCTCGCTTGGAGGGGGAAGTCTCATGGTTTTTCTCGAGTTCC

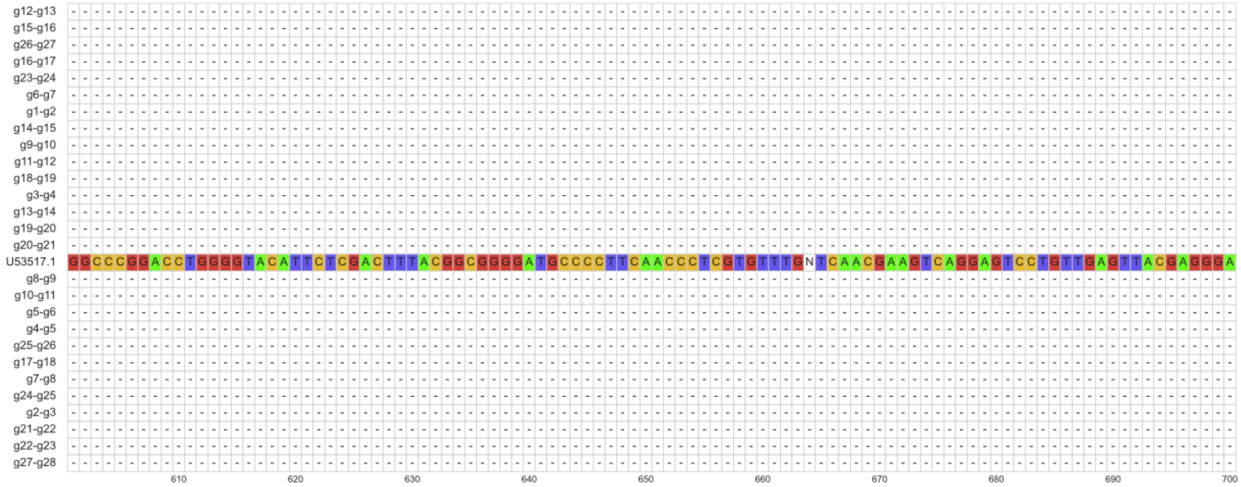

Consensus

GGCCCGGACCTGGGGTACATTCTCGACTTTACGGCGGGGATGCCCCTTCAACCCTCGTGTTTGNTCAACGAAGTCAGGAGTCCTGTTGAGTTACGAGGGA

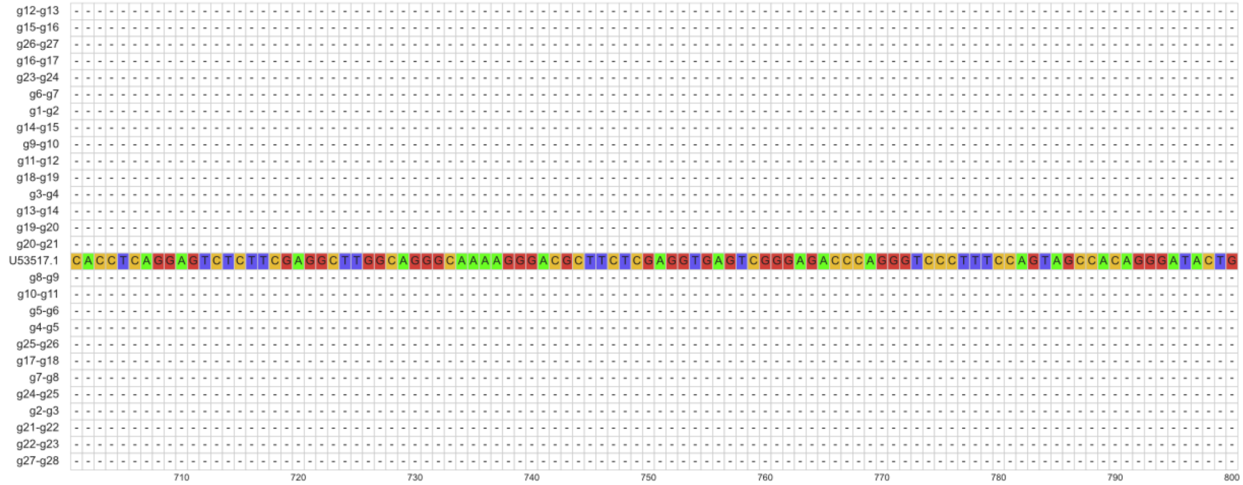

Consensus

CACCTCAGGAGTCTCTTCGAGGCTTGGCAGGGCAAAAGGGACGCTTCTCGAGGTGAGTCGGGAGACCCAGGGTCCCTTTCCAGTAGCCACAGGGATACTG

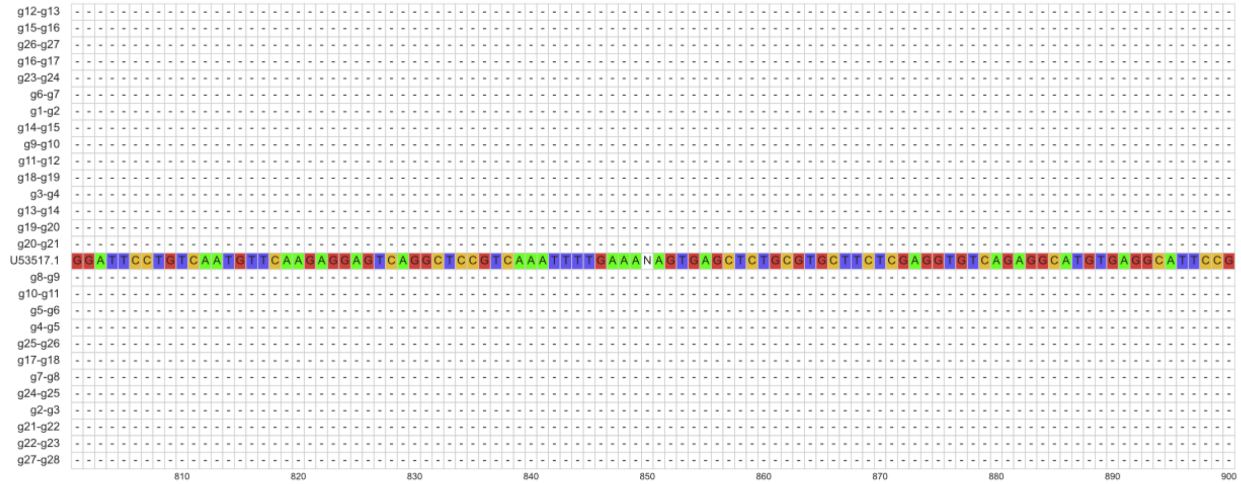

Consensus

GGATTCCTGTCAATGTTCAAGAGGAGTCAGGCTCCGTCAAATTTTGAAANAGTGAGCTCTGCGTGCTTCTCGAGGTGTCAGAGGCATGTGAGGCATTCCG

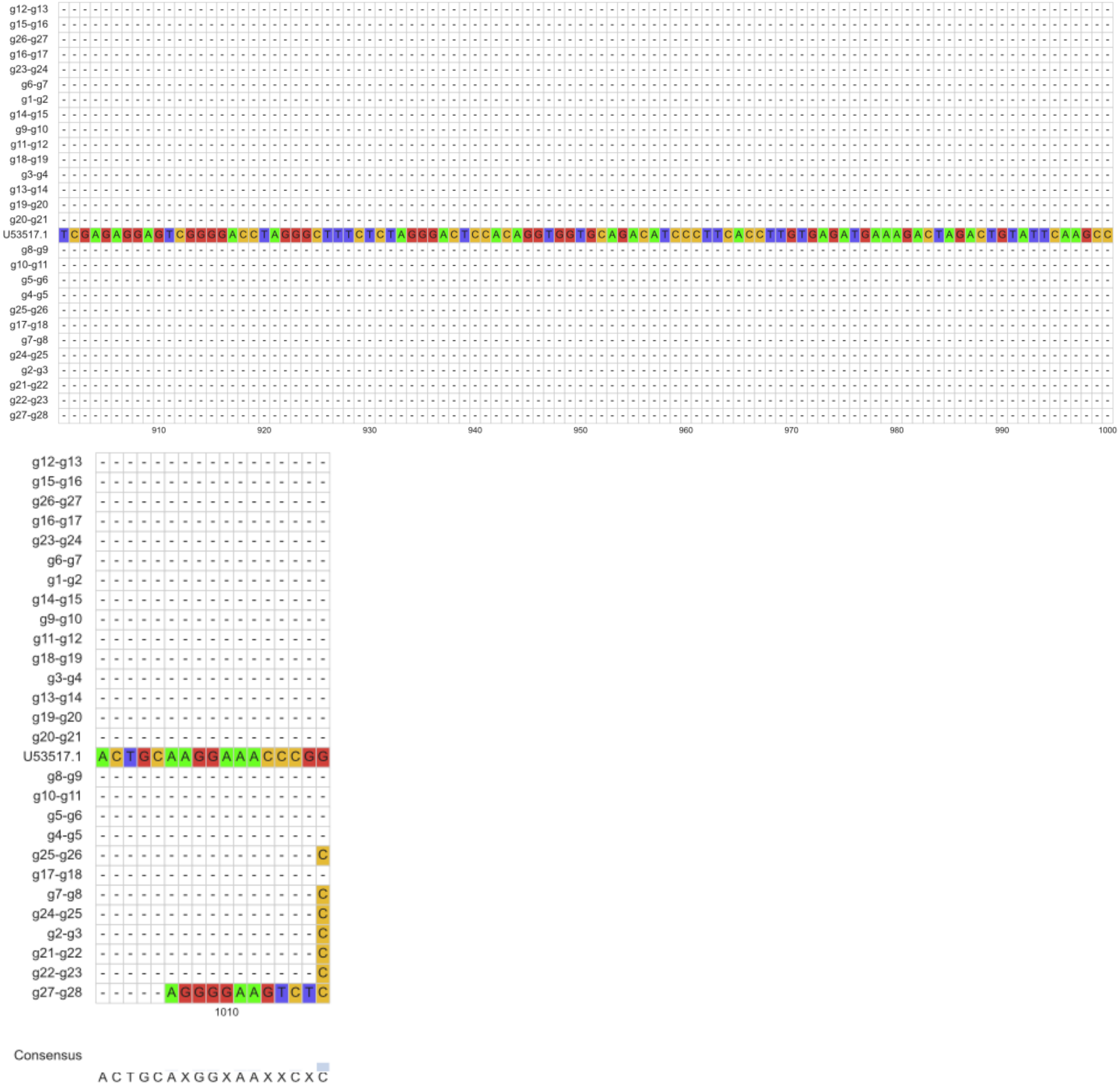
g12-g13
g15-g16
g26-g27
g16-g17
g23-g24
g6-g7
g1-g2
g14-g15
g9-g10
g11-g12
g18-g19
g3-g4
g13-g14
g19-g20
g20-g21
U53517.1
g8-g9
g10-g11
g5-g6
g4-g5
g25-g26
g17-g18
g7-g8
g24-g25
g2-g3
g21-g22
g22-g23
g27-g28
910
920
930
940
950
960
970
980
990
1000
ACTGCAAGGAAACCCGG
AGGGGAAGTCTC
1010
Consensus
A C T G C A X G G X A A X X C X C
